# Supplementary material for: A hierarchical transcriptional network activates specific CDK inhibitors that regulate G2 to control cell size and number in Arabidopsis
Source: Nat Commun. 2022 Mar 29;13:1660. doi: 10.1038/s41467-022-29316-2 (PMC8964727; doi:10.1038/s41467-022-29316-2)
Supplement: Supplementary file 1 — Supplementary Information [file 41467_2022_29316_MOESM1_ESM.pdf]

A hierarchical transcriptional network activates specific CDK inhibitors that regulate G2 to control cell size and number in Arabidopsis

Nomoto, Y. and Takatsuka, H., et al

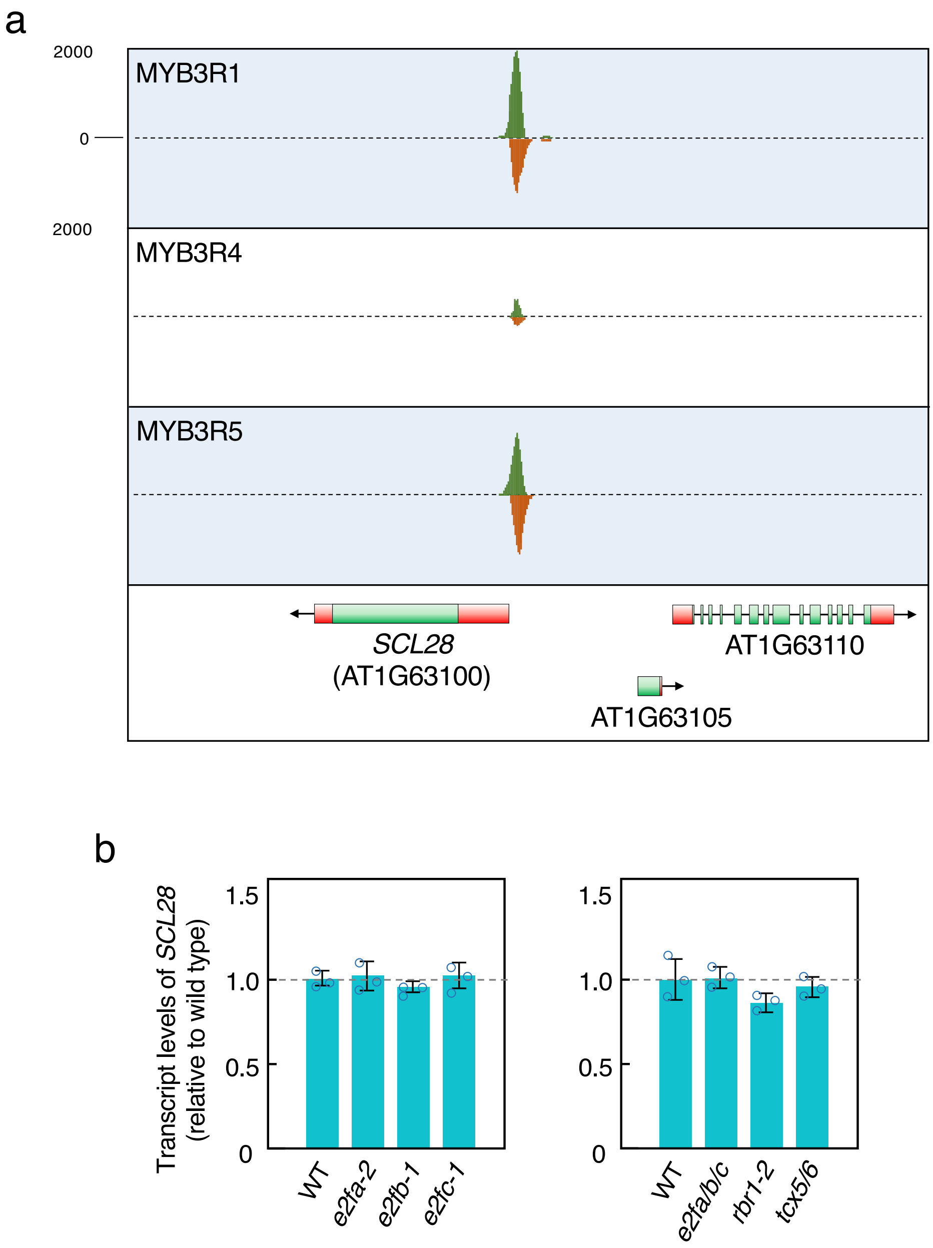

Supplementary Figure 1

Profiles of DAP-seq around *SCL28* locus.

(a) The DAP-seq profiles for MYB3R1, MYB3R4, and MYB3R5 were obtained by the Plant Cistrome Database at http://neomorph.salk.edu/dap_web/pages/index.php (O'Malley et al., 2016). Peaks shown in green and orange correspond to DAP-seq reads of forward and reverse orientations, respectively.

(b) Transcript levels of *SCL28* in the mutants lacking genes for DREAM components. qRT-PCR was performed using whole seedlings of WT, *e2fa-2*, *e2b-1*, and *e2fc-1* (left) and those of WT, *e2fa/b/c*, *rbr1-2*, and *tcx5/6* (right). Data are shown as averages from three biological replicates (± SD). There was no significant difference (two-sided Student's t-test, $P < 0.05$) between WT and any of mutants examined. All mutants and mutant combinations have been described previously (Nowack et al., 2012; Wang et al., 2014; Lang et al., 2121).

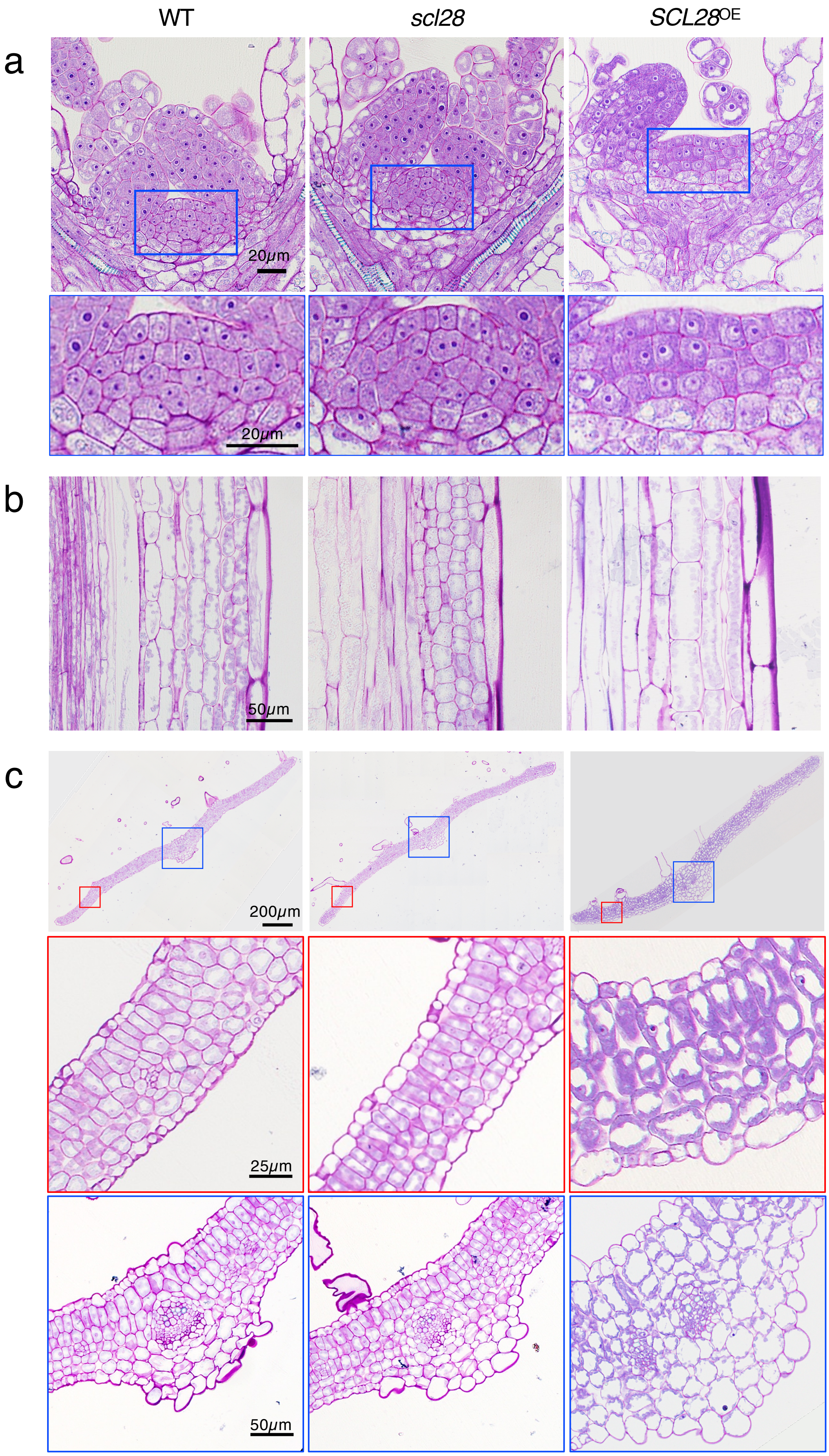

WT
scl28
SCL28OE
a
20μm
20μm
b
50μm
c
200μm
25μm
50μm

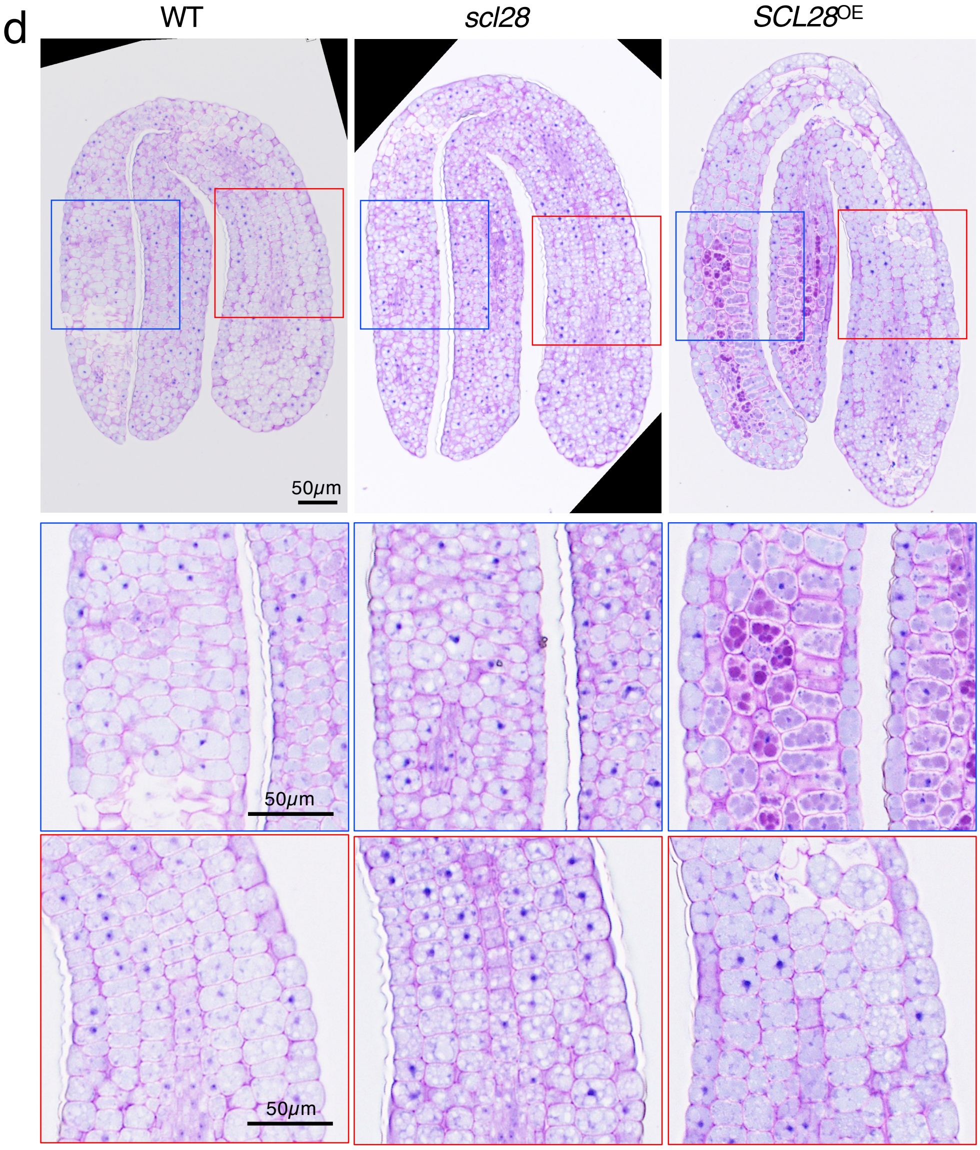

Supplementary Figure 2

Cell size in various organs is oppositely affected in *scl28* and *SCL28*^{OE} plants.

(a) Longitudinal sections of shoot apical meristem and leaf primordia. The area shown in blue rectangles are magnified, and shown in lower panels.

(b) Vertical sections of inflorescence stems.

(c) Transverse sections of leaves. Areas surrounded by red and blue rectangles are magnified and shown in lower panels.

(d) Longitudinal sections of mature embryos. Regions surrounded by red and blue rectangles are magnified and shown in lower panels.

In **(a-d)**, microscopic observations were made for sections from multiple different plants and found each section showing the similar tendency of cell size difference between WT, *scl28*, and *SCL28*^{OE}.

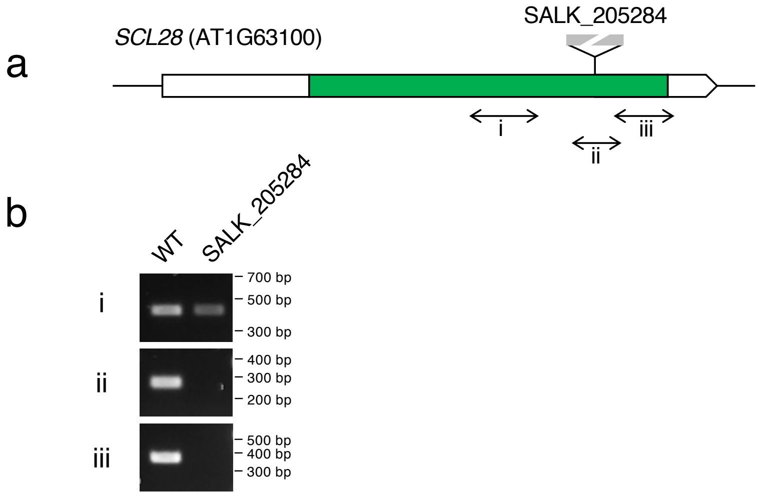

Supplementary Figure 3

Confirmation of T-DNA insertion mutation (SALK_205284) to be a null allele of *SCL28*.

(**a**) Position of T-DNA insertion in the *SCL28* gene. Exons are shown by boxes, and introns by lines between boxes, where green and white boxes indicate coding and non-coding regions, respectively. Amplified regions by semi-quantitative PCR analysis are shown by double-headed arrows.

(**b**) Semi-quantitative PCR was performed using primer pairs sandwiching the regions (i, ii, and iii) indicated in (**a**). Multiple individual plants were analyzed with similar results. Positions of the size markers are shown on the right.

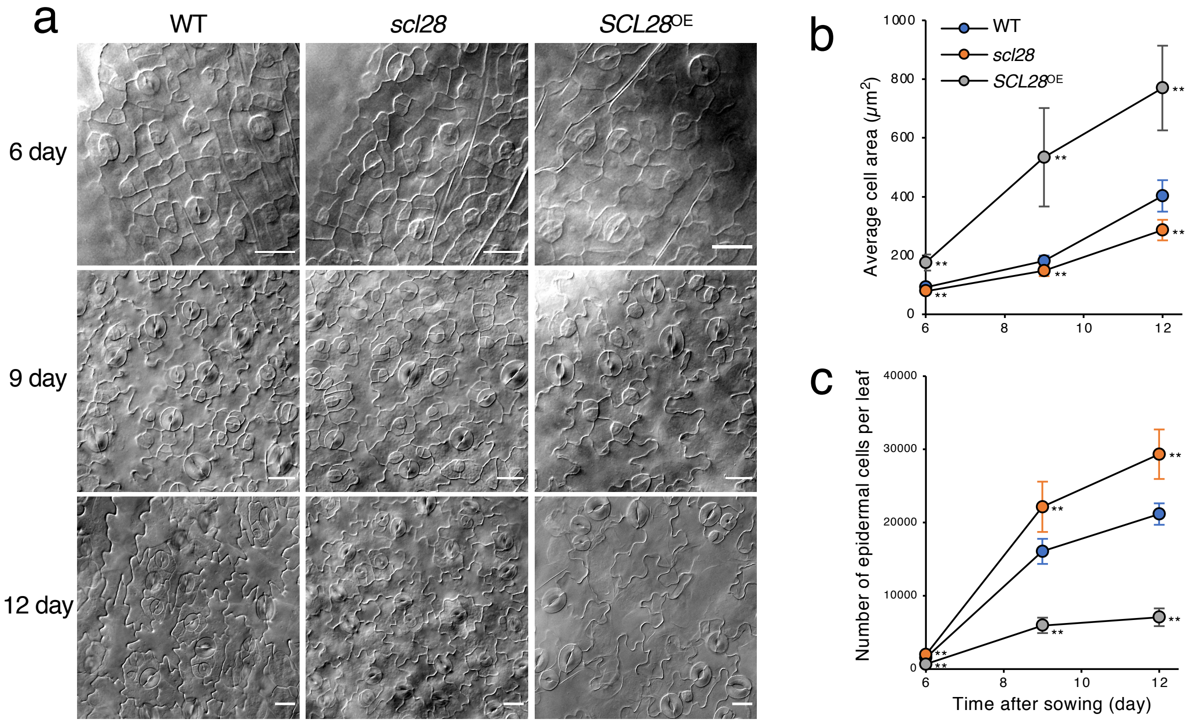

Supplementary Figure 4

Kinematic analysis of leaf growth focusing on epidermal cells in WT, *scl28*, and *SCL28*^{OE} plants.

(**a**) Images of abaxial epidermis from WT, *scl28*, and *SCL28*^{OE} plants. First leaf pairs from plants at 6, 9 and 12 DAS were cleared and analyzed by DIC microscopy. Scale bar indicates 20 μm.

(**b-c**) Quantitative analysis of epidermal cell area (**a**) and number of epidermal cells per leaf (**b**) in abaxial epidermis of developing first leaf pairs. Values are averages from the data of 10 different plants (± SD), in each of which more than 65 cells were analyzed. Statistical significance was determined using two-sided Student's t-test. $^{**}P < 0.01$.

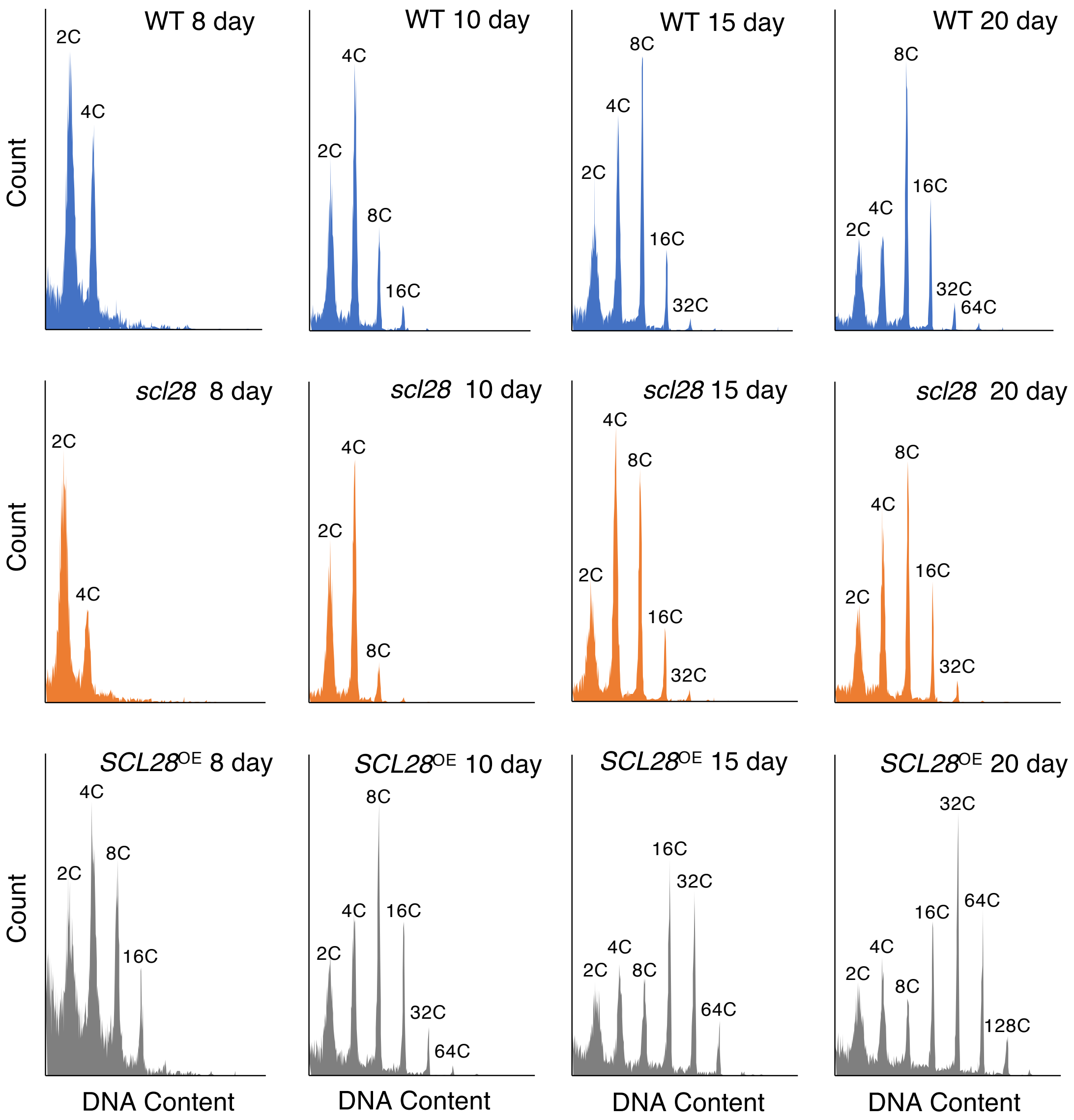

Supplementary Figure 5

Ploidy analysis of WT, *scl28*, and *SCL28*^{OE} plants.

Representative profiles of ploidy distribution in first leaf pairs from WT, *scl28*, and *SCL28*^{OE} plants grown for indicated period after sowing.

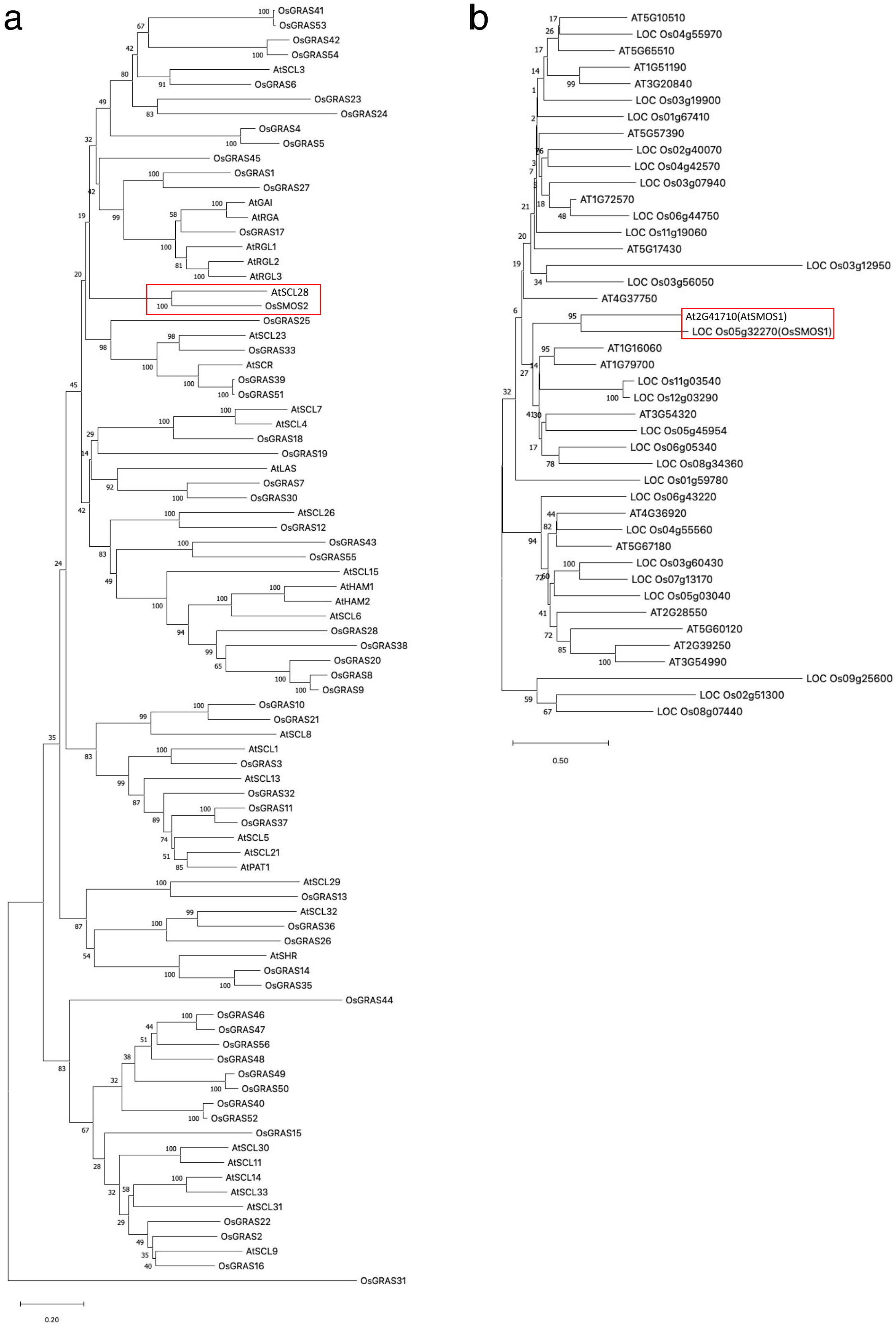

Supplementary Figure 6

Phylogenetic analysis of SCL28 and AtSMOS1

(**a**) Phylogenetic analysis of GRAS family proteins from rice and *Arabidopsis*, showing that SCL28 is orthologous to rice SMOS2. Amino acid sequences of all GRAS proteins in rice and *Arabidopsis* were

obtained in Phytozome at https://phytozome.jgi.doe.gov/pz/portal.html (Goodstein et al., 2012). Amino acid sequence alignment within GRAS domain was generated by the MUSCLE program (Edgar 2004), and used for creating phylogenetic trees using MEGAX (Kumar et al. 2018) based on neighbor-joining method (Saitou and Nei, 1987).

(b) Phylogenetic analysis of AP2-type transcription factors from rice and Arabidopsis, showing that At2g41710 (AtSMOS1) is orthologous to rice SMOS1. Amino acid sequences of Arabidopsis proteins categorized as AP2 subfamily (Dietz et al., 2010) and corresponding rice proteins (Sharoni et al., 2011) were obtained and analyzed as in (**a**).

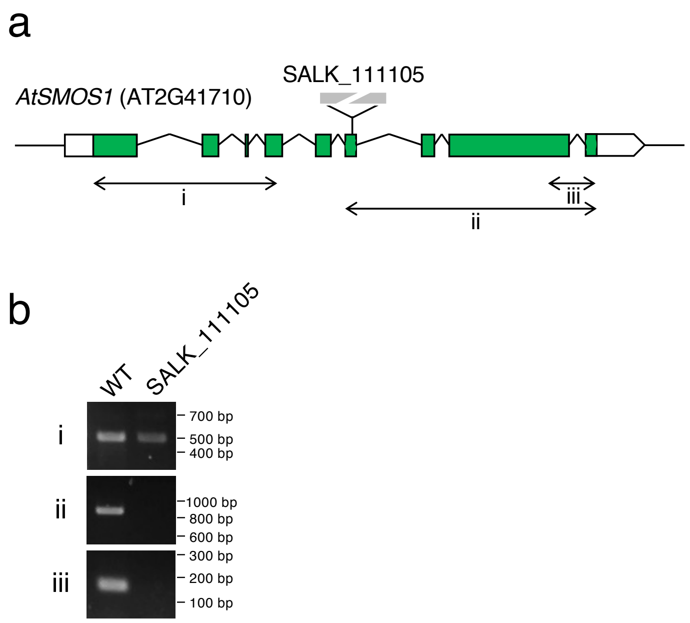

Supplementary Figure 7

Confirmation of T-DNA insertion mutation (SALK_111105) to be a null allele of *AtSMOS1*.

(**a**) Structure of *AtSMOS1* gene (At2g41710) and the position of T-DNA insertion. Exons are shown by boxes and introns by lines between boxes, where green and white boxes indicate coding and non-coding regions, respectively. Amplified regions by semi-quantitative PCR analysis are shown by double-headed arrows.

(b) Semi-quantitative PCR was performed using primer pairs sandwiching the regions (i, ii, and iii) indicated in (**a**). Multiple individual plants were analyzed with similar results. Positions of the size markers are shown on the right.

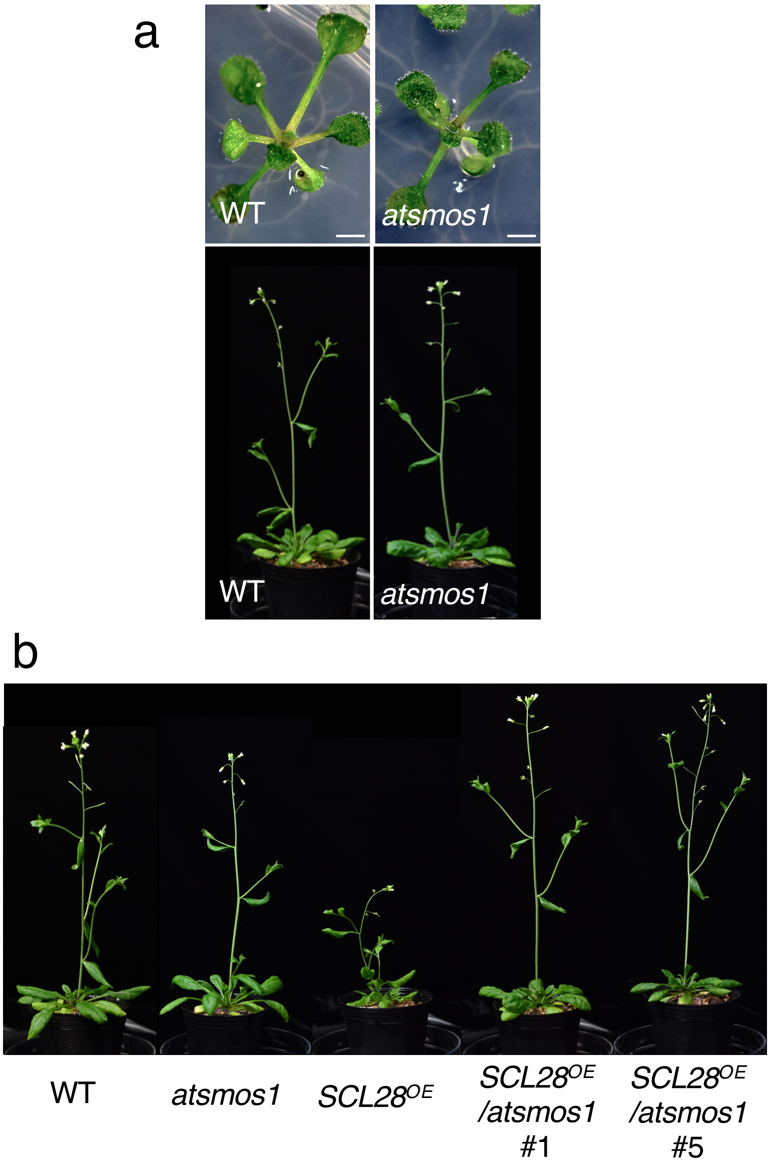

Supplementary Figure 8

***atsmos1* mutation does not significantly affect the plant growth, but completely suppress the growth inhibition caused by *SCL28* overexpression**

(a) Comparison of whole plant appearance between WT and *atsmos1*. Plants grown for 12 days on agar medium (upper) and those grown for four weeks on soil (lower) were photographed. Scale bars indicate 1 mm in upper panels.

(b) Comparison of whole plant appearance between WT, *atsmos1*, and *SCL28*^{OE} plants and those carrying *atsmos1* and *SCL28*^{OE} in combination (*SCL28*^{OE}/ *atsmos1*). Plants with indicated genotypes were photographed four weeks after sowing. Two independent lines of *SCL28*^{OE}/ *atsmos1* were analyzed.

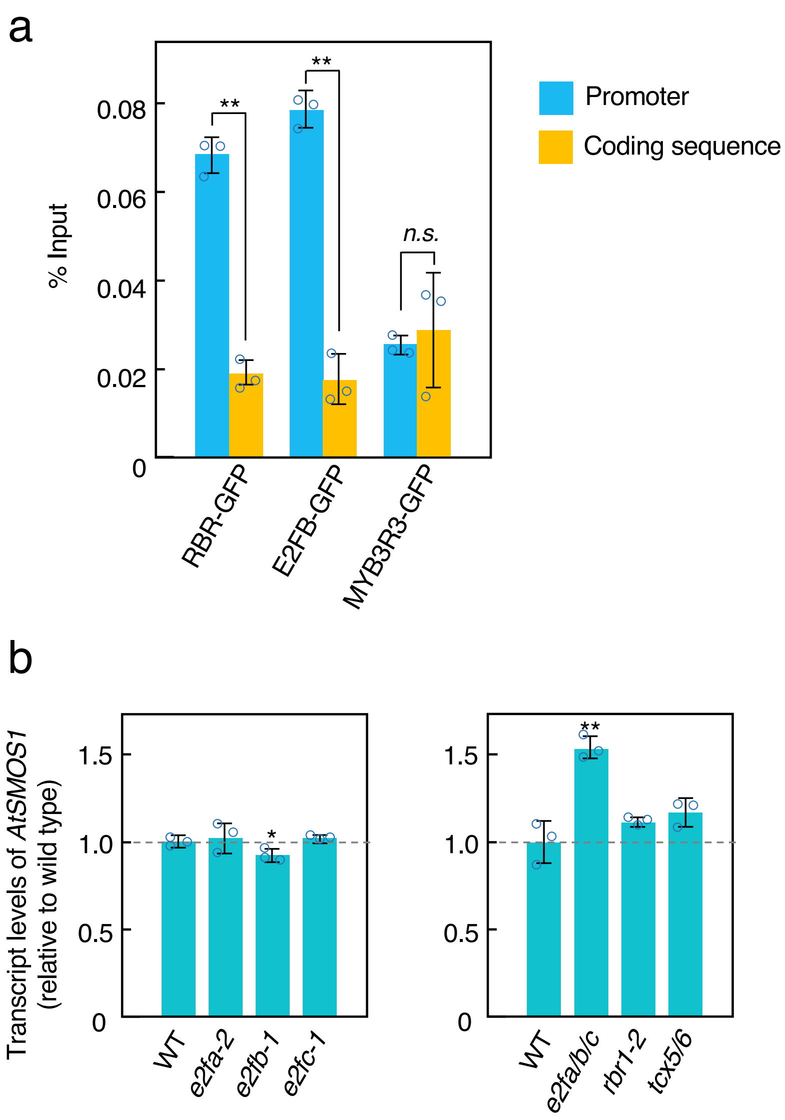

Supplementary Figure 9

***AtSMOS1* may be a downstream target regulated by E2F-RBR pathway**

(a) ChIP–qPCR assays were performed using whole seedlings carrying proRBR::RBR-GFP, proE2FB::E2FB-GFP, or proMYB3R3::MYB3R3-GFP. Amount of DNA fragments co-immunoprecipitated with anti-GFP antibody was determined at both promoter region and CDS of *AtSMOS1,* and percentage of ChIPed DNA relative to input DNA was calculated. Data are shown as average from three technical replicates (± SD). Statistical significance was determined using two-sided Student's t-test. ** $P < 0.01$. *n.s.*, not significant. All transgenic lines carrying GFP fusion constructs were described previously (Magyar et al., 2012; Őszi et al., 2020; Kobayashi et al., 2015).

(b) Transcript levels of *AtSMOS1* in the mutants lacking genes for DREAM components. qRT-PCR was performed using whole seedlings of WT, *e2fa-2*, *e2b-1*, and *e2fc-1* (left) and those of WT, *e2fa/b/c*, *rbr1-2*, and *tcx5/6* (right). Data are shown as averages from three biological replicates (± SD). Statistical significance was determined using two-sided Student's t-test. *$P < 0.05$, **$P < 0.01$. All mutants and mutant combinations have been described previously (Nowack et al., 2012; Wang et al., 2014; Lang et al., 2121).

a

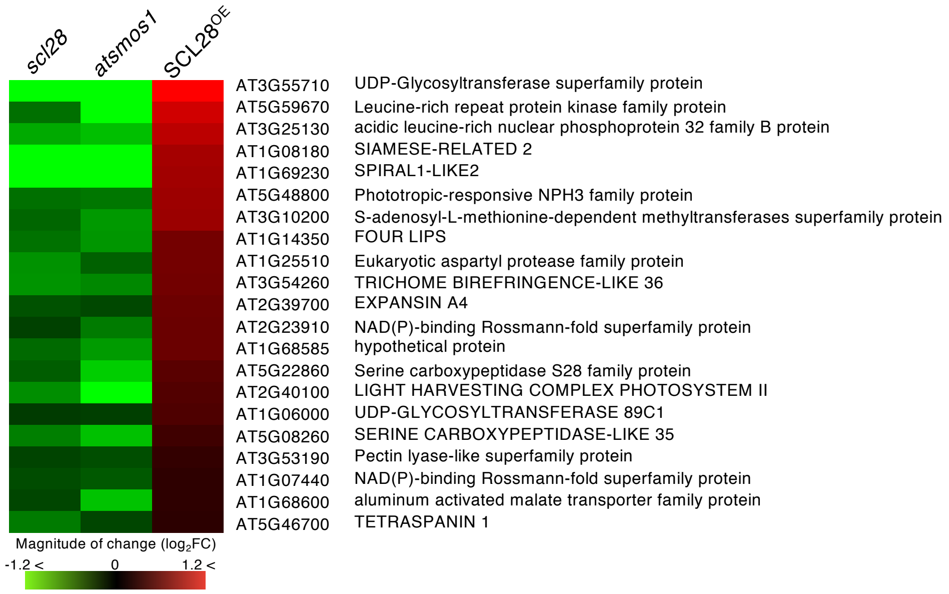

b

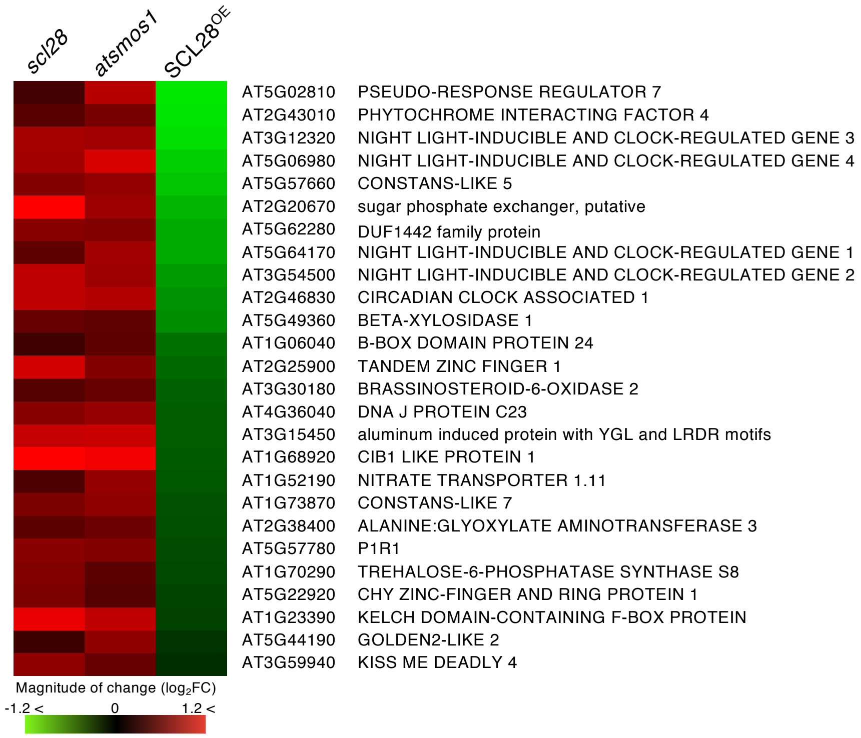

Supplementary Figure 10

Heatmap representation of candidate genes regulated by SCL28-AtSMOS1 complex.

(a) Heatmap showing expression changes of 21 genes downregulated in both *scl28* and *atsmos1* and upregulated in *SCL28*^{OE}.

(b) Heatmap showing expression changes of 26 genes upregulated in both *scl28* and *atsmos1* and downregulated in *SCL28*^{OE}.

Fold change level (log2) of each gene was calculated by comparing expression levels in *scl28*, *atsmos1*, or *SCL28*^{OE} plants with that in WT plants.

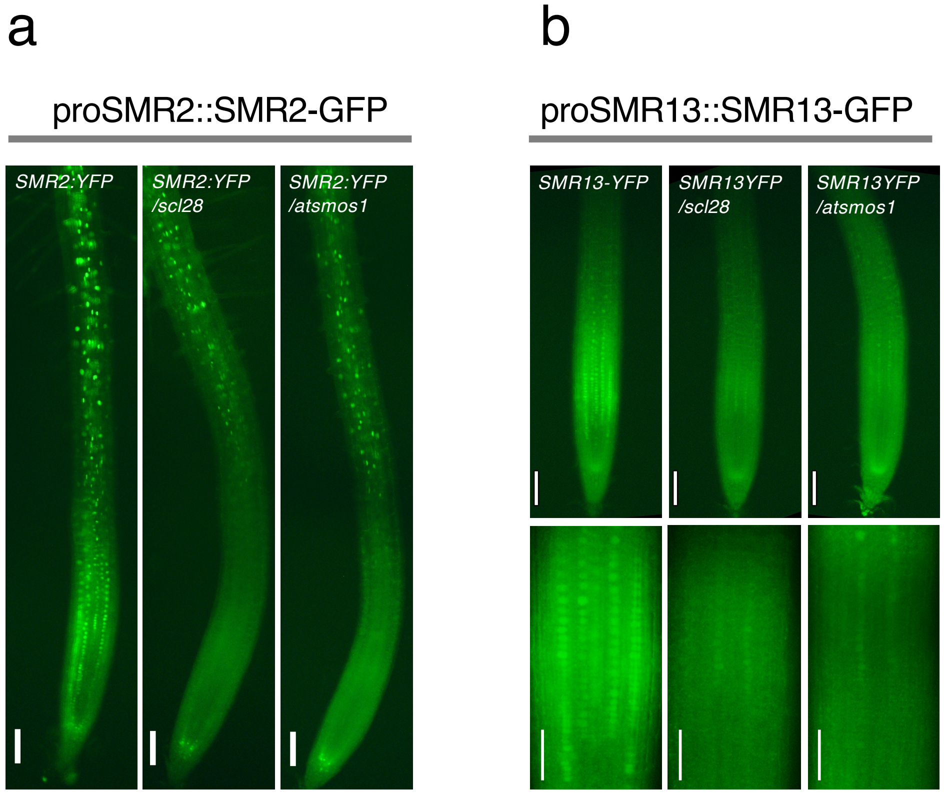

Supplementary Figure 11

Downregulation of SMR2-GFP and SMR13-GFP in *scl28* and *atsmos1* mutants.

(a) Fluorescent images of primary roots from plants at 7 DAS carrying proSMR2::SMR2-GFP under WT, *scl28* or *atsmos1* background. Scale bars indicate 100 μm

(b) Fluorescent images of primary roots from plants at 7 DAS carrying proSMR13::SMR13-GFP under WT, *scl28* or *atsmos1* background. Magnified views of meristematic regions are shown in lower panels. Scale bars indicate 100 μm (upper) and 50 μm (lower).

In **(a)** and **(b)**, similar difference in GFP expression between WT, *scl28* and *atsmos1* was confirmed in multiple independent lines.

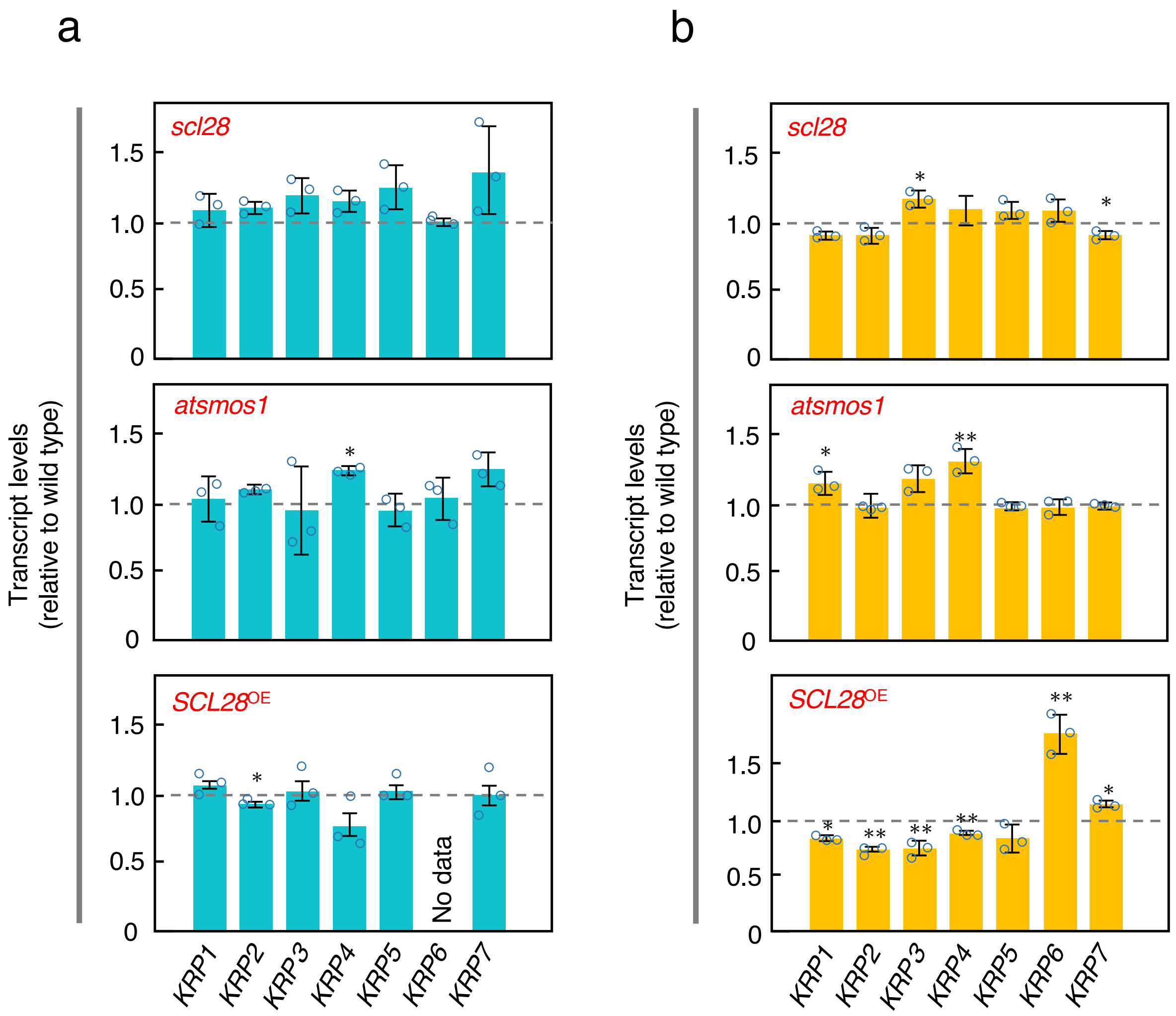

Supplementary Figure 12

Transcript levels of *KRP* genes are not largely affected by *SCL28* and *AtSMOS1*.

(a) Expression data of each *KRP* gene in *scl28*, *atsmos1*, and *SCL28*^{OE} plants were collected from transcriptome data, and the relative expression level in each line was calculated by comparing with WT.

(b) Expression levels in *scl28*, *atsmos1*, and *SCL28*^{OE} plants were validated for each *KRP* gene by qRT-PCR analysis. Relative expression level in each line was calculated by comparing with WT.

In **(a)** and **(b)**, data are shown as averages from three biological replicates (± SD). Statistical significance compared with WT was determined using two-sided Student’s t-test. *$P < 0.05$, **$P < 0.01$

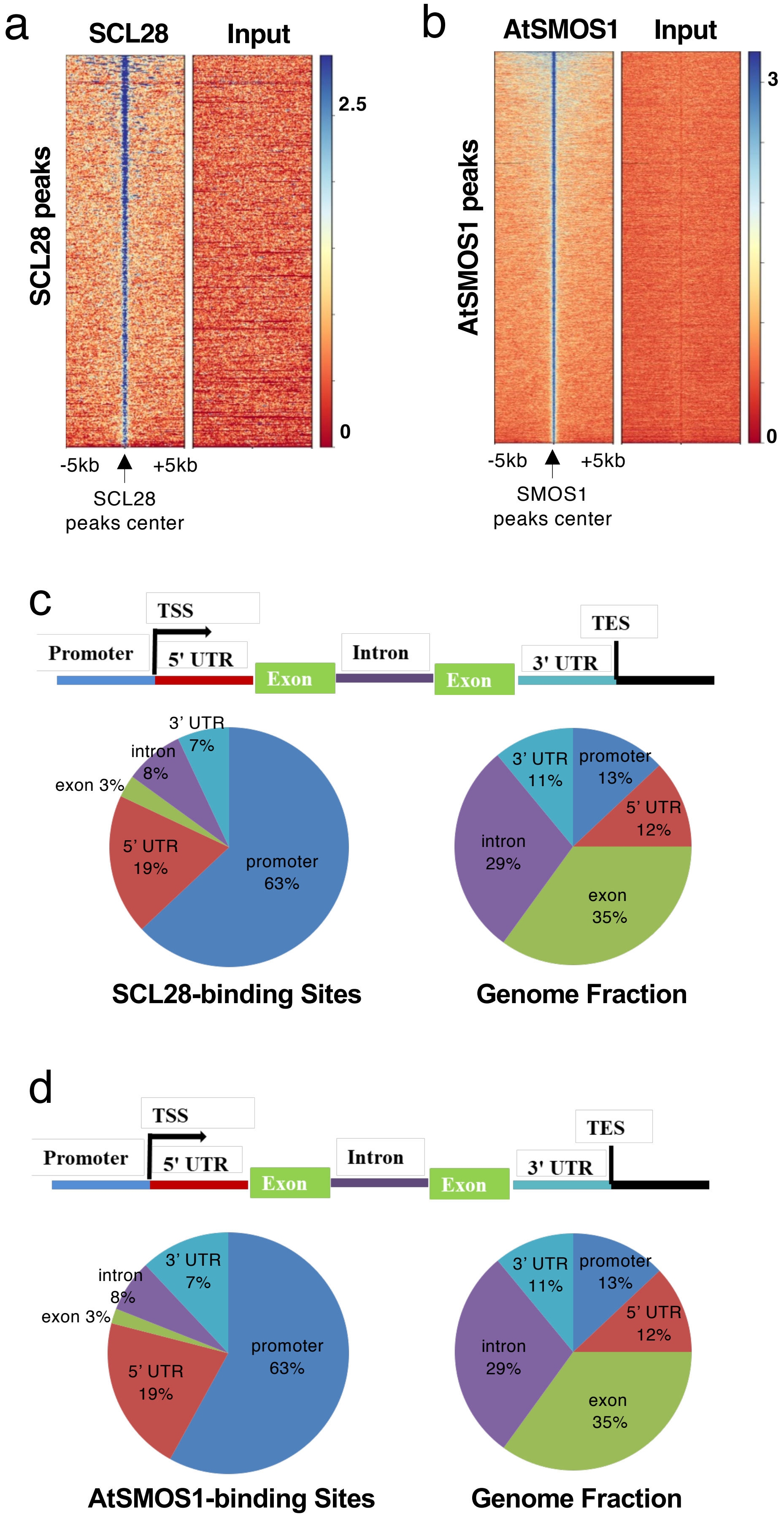

a
SCL28
Input
SCL28 peaks
2.5
0
-5kb
+5kb
SCL28
peaks center
b
AtSMOS1
Input
AtSMOS1 peaks
3
0
-5kb
+5kb
SMOS1
peaks center
c
TSS
TES
Promoter
5' UTR
Exon
Intron
Exon
3' UTR
3' UTR 7%
intron 8%
exon 3%
5' UTR 19%
promoter 63%
3' UTR 11%
promoter 13%
5' UTR 12%
intron 29%
exon 35%
SCL28-binding Sites
Genome Fraction
d
TSS
TES
Promoter
5' UTR
Exon
Intron
Exon
3' UTR
3' UTR 7%
intron 8%
exon 3%
5' UTR 19%
promoter 63%
3' UTR 11%
promoter 13%
5' UTR 12%
intron 29%
exon 35%
AtSMOS1-binding Sites
Genome Fraction

Supplementary Figure 13

SCL28 and AtSMOS1 preferentially bind to promoter regions.

(a) Comparison between SCL28 and input of tag density in the ±5 kb region around the SCL28 peaks showing ChIP-Seq peaks of SCL28 were successfully detected.

(b) Comparison between AtSMOS1 and input of tag density in the ±5 kb region around the AtSMOS1 peaks showing ChIP-Seq peaks of AtSMOS1 were successfully detected.

(c) Pie chart representation of the distribution of SCL28 peaks identified by ChIP-Seq in different genomic regions. The definition of each region is described above the pie chart.

(d) Pie chart representation of the distribution of AtSMOS1 peaks identified by ChIP-Seq in different genomic regions. The definition of each region is described above the pie chart.

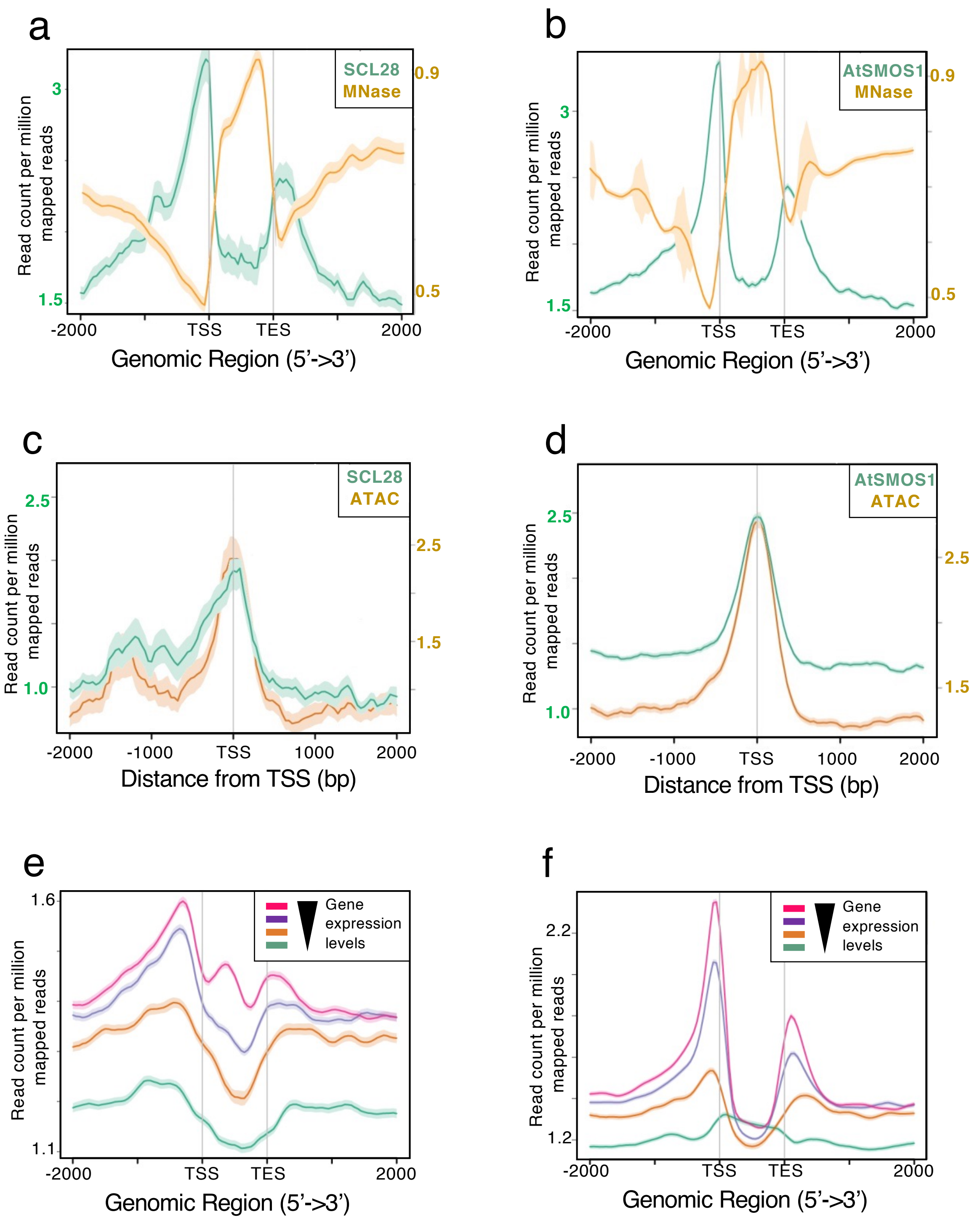

Supplementary Figure 14

SCL28 and AtSMOS1 bind to nucleosome-free and highly accessible chromatin regions with their enrichment levels associated with mRNA levels

(a) SCL28 binds nucleosome-free regions of transcribed genes. Mean profile of SCL28 ChIP-Seq and MNase-seq reads density with respect to a gene model from TSS to TES. Normalization of coverage using

spline algorithm was performed over the genes and flanking 2 kb region.

(b) AtSMOS1 binds nucleosome-free regions of transcribed genes. Data from AtSMOS1 ChIP-Seq and MNase-seq were analyzed and shown as in (**a**).

(c) SCL28 binds to chromatin accessible sites. Profiles of SCL28 ChIP-Seq and ATAC-seq reads density were merged over TSS and flanking 2 kb region.

(d) AtSMOS1 binds to chromatin accessible sites. Profiles of AtSMOS1 ChIP-Seq and ATAC-seq reads density were merged over TSS and flanking 2 kb region.

(e) Highly-expressed genes show higher enrichment for SCL28 binding. Average enrichment profile of SCL28 is correlated with gene expression variations. Gene expression is categorized from low to high expression. Mean-normalized ChIP-Seq densities of equal bins were plotted along the gene and 2-kb region flanking the TSS or TES.

(f) Highly-expressed genes show higher enrichment for AtSMOS1 binding. ChIP-Seq data of AtSMOS1 were analyzed as in (**e**).

In **(a)**-**(f)**, solid line represents median signal and lighter shading represents standard error and 95% confidence interval.

	GO biological process	Arabidopsis thaliana REFLIST	#	Expected	over/ under	Fold Enrichment	raw P-value	FDR
●	negative regulation of mitotic nuclear division (GO:0045839)	25	5	0.17	+	28.76	1.85E-06	5.50E-03
●	negative regulation of nuclear division (GO:0051784)	30	5	0.21	+	23.96	4.09E-06	4.87E-03
●	regulation of mitotic nuclear division (GO:0007088)	36	5	0.25	+	19.97	9.14E-06	9.06E-03
●	regulation of DNA endoreduplication (GO:0032875)	37	5	0.26	+	19.43	1.03E-05	8.76E-03
	water transport (GO:0006833)	30	4	0.21	+	19.17	8.70E-05	3.04E-02
	fluid transport (GO:0042044)	30	4	0.21	+	19.17	8.70E-05	2.87E-02
●	regulation of DNA-dependent DNA replication (GO:0090329)	47	5	0.33	+	15.3	2.98E-05	2.22E-02
●	negative regulation of mitotic cell cycle (GO:0045930)	48	5	0.33	+	14.98	3.27E-05	2.16E-02
●	regulation of nuclear division (GO:0051783)	50	5	0.35	+	14.38	3.92E-05	2.33E-02
	negative regulation of organelle organization (GO:0010639)	50	5	0.35	+	14.38	3.92E-05	2.12E-02
●	regulation of DNA replication (GO:0006275)	55	5	0.38	+	13.07	5.99E-05	2.37E-02
●	negative regulation of cell cycle process (GO:0010948)	68	5	0.47	+	10.57	1.53E-04	4.14E-02
●	regulation of mitotic cell cycle (GO:0007346)	86	6	0.6	+	10.03	4.37E-05	2.17E-02
	response to water (GO:0009415)	376	11	2.62	+	4.21	8.55E-05	3.18E-02
	response to acid chemical (GO:0001101)	408	11	2.84	+	3.88	1.71E-04	4.43E-02
	response to oxygen-containing compound (GO:1901700)	1431	28	9.95	+	2.81	9.52E-07	5.67E-03
	oxoacid metabolic process (GO:0043436)	976	18	6.79	+	2.65	1.96E-04	4.66E-02
	cellular response to chemical stimulus (GO:0070887)	1117	20	7.77	+	2.57	1.26E-04	3.96E-02
	response to external stimulus (GO:0009605)	1527	24	10.62	+	2.26	2.02E-04	4.62E-02
	response to chemical (GO:0042221)	2530	39	17.6	+	2.22	2.26E-06	4.49E-03
	regulation of cellular macromolecule biosynthetic process (GO:2000112)	2374	33	16.51	+	2	1.42E-04	4.21E-02
	regulation of macromolecule biosynthetic process (GO:0010556)	2387	33	16.6	+	1.99	1.51E-04	4.28E-02
	regulation of cellular biosynthetic process (GO:0031326)	2488	34	17.3	+	1.96	1.94E-04	4.82E-02
	regulation of biosynthetic process (GO:0009889)	2525	34	17.56	+	1.94	2.17E-04	4.78E-02
	response to stress (GO:0006950)	3187	42	22.17	+	1.89	5.70E-05	2.42E-02
	response to stimulus (GO:0050896)	5474	66	38.07	+	1.73	2.73E-06	4.06E-03
	cellular process (GO:0009987)	12044	112	83.77	+	1.34	5.16E-05	2.36E-02

● Cell cycle-related GO terms

1 Fold change 20

Supplementary Figure 15

Gene ontology enrichment analysis of common targets of SCL28 and AtSMOS1.

GO enrichment analysis was performed using PANTHER overrepresentation test with the "GO biological processes complete" dataset. Overrepresented GO terms related to cell cycle are shown by red dots on the left. Blue backgrounds show the number of common target genes belonging to the corresponding cell cycle-related GO categories, all of which include five common *SMR* genes (*SMR2*, *SMR4*, *SMR6*, *SMR8*, and *SMR9*). Enrichment of GO terms was analyzed using one-sided Fisher exact test, and its statistical significance was evaluated by raw *P*-value and FDR-adjusted *P*-value.

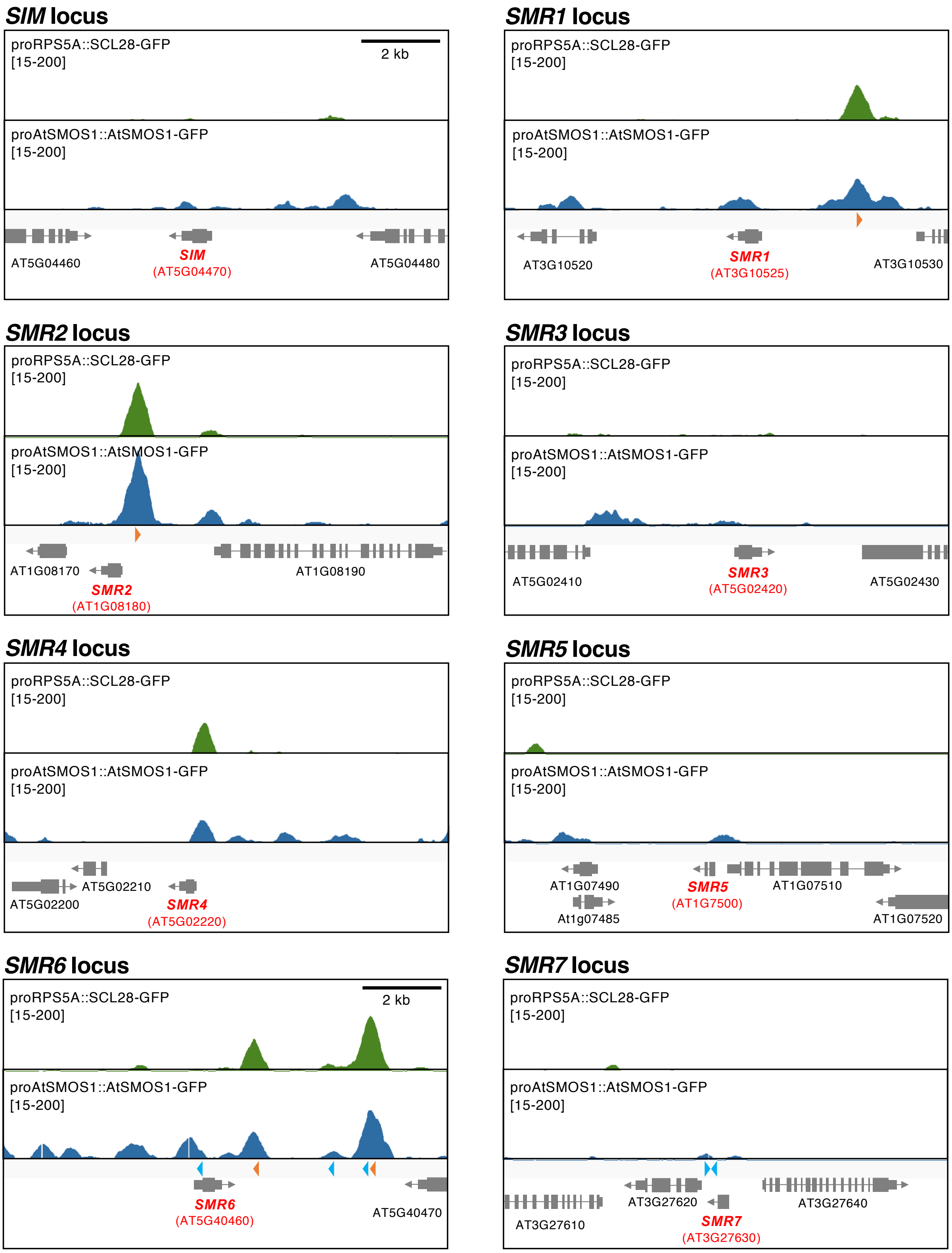

SIM locus
proRPS5A::SCL28-GFP
[15-200]
2 kb
proAtSMOS1::AtSMOS1-GFP
[15-200]
AT5G04460
SIM
(AT5G04470)
AT5G04480
SMR1 locus
proRPS5A::SCL28-GFP
[15-200]
proAtSMOS1::AtSMOS1-GFP
[15-200]
AT3G10520
SMR1
(AT3G10525)
AT3G10530
SMR2 locus
proRPS5A::SCL28-GFP
[15-200]
proAtSMOS1::AtSMOS1-GFP
[15-200]
AT1G08170
SMR2
(AT1G08180)
AT1G08190
SMR3 locus
proRPS5A::SCL28-GFP
[15-200]
proAtSMOS1::AtSMOS1-GFP
[15-200]
AT5G02410
SMR3
(AT5G02420)
AT5G02430
SMR4 locus
proRPS5A::SCL28-GFP
[15-200]
proAtSMOS1::AtSMOS1-GFP
[15-200]
AT5G02210
AT5G02200
SMR4
(AT5G02220)
SMR5 locus
proRPS5A::SCL28-GFP
[15-200]
proAtSMOS1::AtSMOS1-GFP
[15-200]
AT1G07490
SMR5
(AT1G7500)
AT1G07510
At1g07485
AT1G07520
SMR6 locus
proRPS5A::SCL28-GFP
[15-200]
2 kb
proAtSMOS1::AtSMOS1-GFP
[15-200]
SMR6
(AT5G40460)
AT5G40470
SMR7 locus
proRPS5A::SCL28-GFP
[15-200]
proAtSMOS1::AtSMOS1-GFP
[15-200]
AT3G27620
AT3G27640
AT3G27610
SMR7
(AT3G27630)

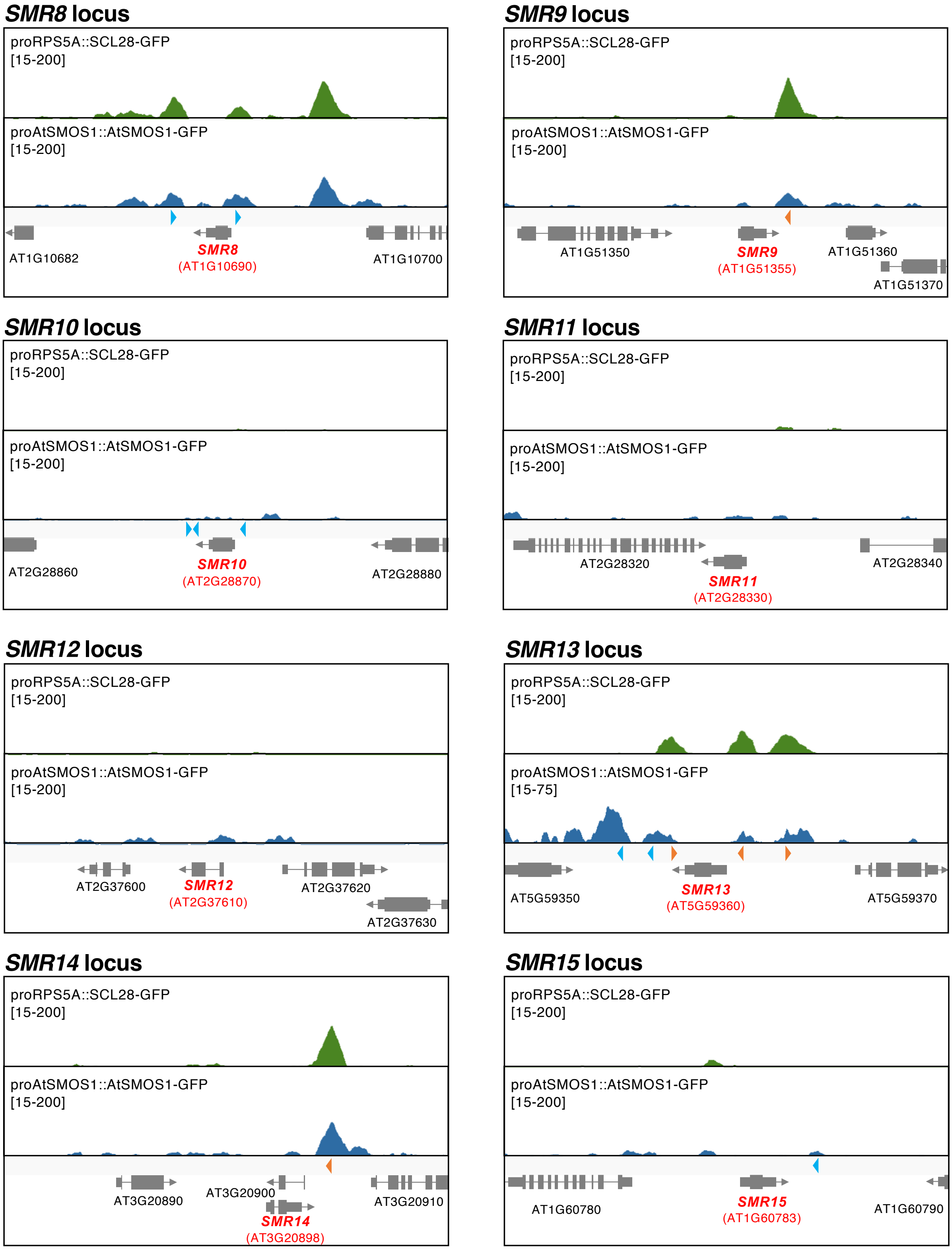
SMR8 locus
proRPS5A::SCL28-GFP
[15-200]
proAtSMOS1::AtSMOS1-GFP
[15-200]
AT1G10682
SMR8
(AT1G10690)
AT1G10700
SMR9 locus
proRPS5A::SCL28-GFP
[15-200]
proAtSMOS1::AtSMOS1-GFP
[15-200]
AT1G51350
SMR9
(AT1G51355)
AT1G51360
AT1G51370
SMR10 locus
proRPS5A::SCL28-GFP
[15-200]
proAtSMOS1::AtSMOS1-GFP
[15-200]
AT2G28860
SMR10
(AT2G28870)
AT2G28880
SMR11 locus
proRPS5A::SCL28-GFP
[15-200]
proAtSMOS1::AtSMOS1-GFP
[15-200]
AT2G28320
SMR11
(AT2G28330)
AT2G28340
SMR12 locus
proRPS5A::SCL28-GFP
[15-200]
proAtSMOS1::AtSMOS1-GFP
[15-200]
AT2G37600
SMR12
(AT2G37610)
AT2G37620
AT2G37630
SMR13 locus
proRPS5A::SCL28-GFP
[15-200]
proAtSMOS1::AtSMOS1-GFP
[15-75]
AT5G59350
SMR13
(AT5G59360)
AT5G59370
SMR14 locus
proRPS5A::SCL28-GFP
[15-200]
proAtSMOS1::AtSMOS1-GFP
[15-200]
AT3G20890
AT3G20900
SMR14
(AT3G20898)
AT3G20910
SMR15 locus
proRPS5A::SCL28-GFP
[15-200]
proAtSMOS1::AtSMOS1-GFP
[15-200]
AT1G60780
SMR15
(AT1G60783)
AT1G60790

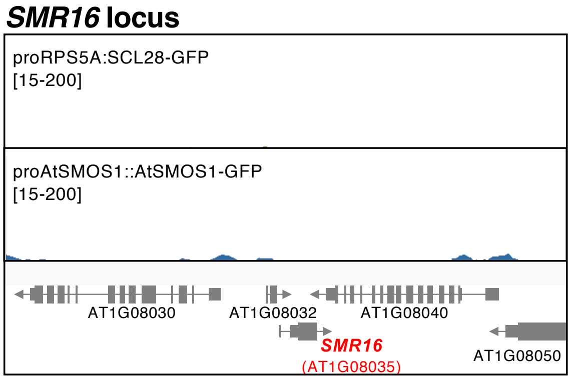

Supplementary Figure 16

ChIP-Seq profile of SCL28 and AtSMOS1 around the *SMR* genes.

Genome-wide binding of SCL28 and AtSMOS1 was analyzed by ChIP-Seq using proRPS5A::SCL28-GFP and proAtSMOS1::AtSMOS1-GFP plants. The ChIP-Seq profiles of SCL28 and AtSMOS1 are shown for all 17 genes of the SMR family in Arabidopsis. Orange arrowheads indicate DNA motifs perfectly matching C(a/t)T(a/t)GGATNC(c/t)(a/t) identified as an enriched motif in the SCL28/AtSMOS1 common targets, whereas blue arrowheads indicate motifs matching the enriched motif with one base mismatch.

a

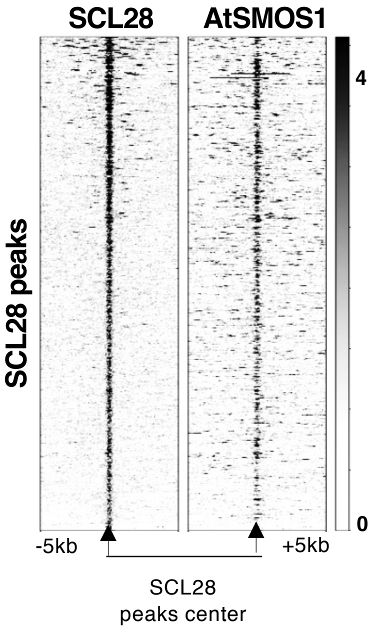

b

Motif	*P*-value	log *P*-value
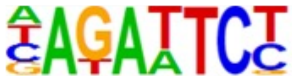	1e-31	-7.142e+01
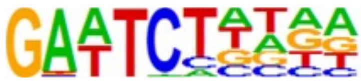	1e-27	-6.327e+01
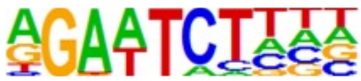	1e-26	-6.107e+01

c

Motif	*P*-value	log *P*-value
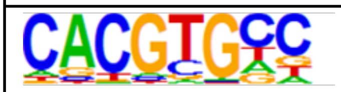	1e-241	-5.553e+02

Supplementary Figure 17

Supporting evidence for binding of SCL28 and AtSMOS1 together at the same genomic sites.

(a) Co-occurrence of ChIP-Seq peak of SCL28 and AtSMOS1 at the genome-wide scale. SCL28 and AtSMOS1 tag density was compared in the ±5 kb region around the SCL28 peaks.

(b) Motifs enriched in SCL28 targets. HOMER motif search identified major SCL28-associated motifs.

(c) Motifs enriched in AtSMOS1 targets. HOMER motif search identifies major AtSMOS1-associated motif.

In **(b)** and **(c),** motif enrichment was evaluated by *P*-value from one-sided hypergeometric tests (no adjustment).

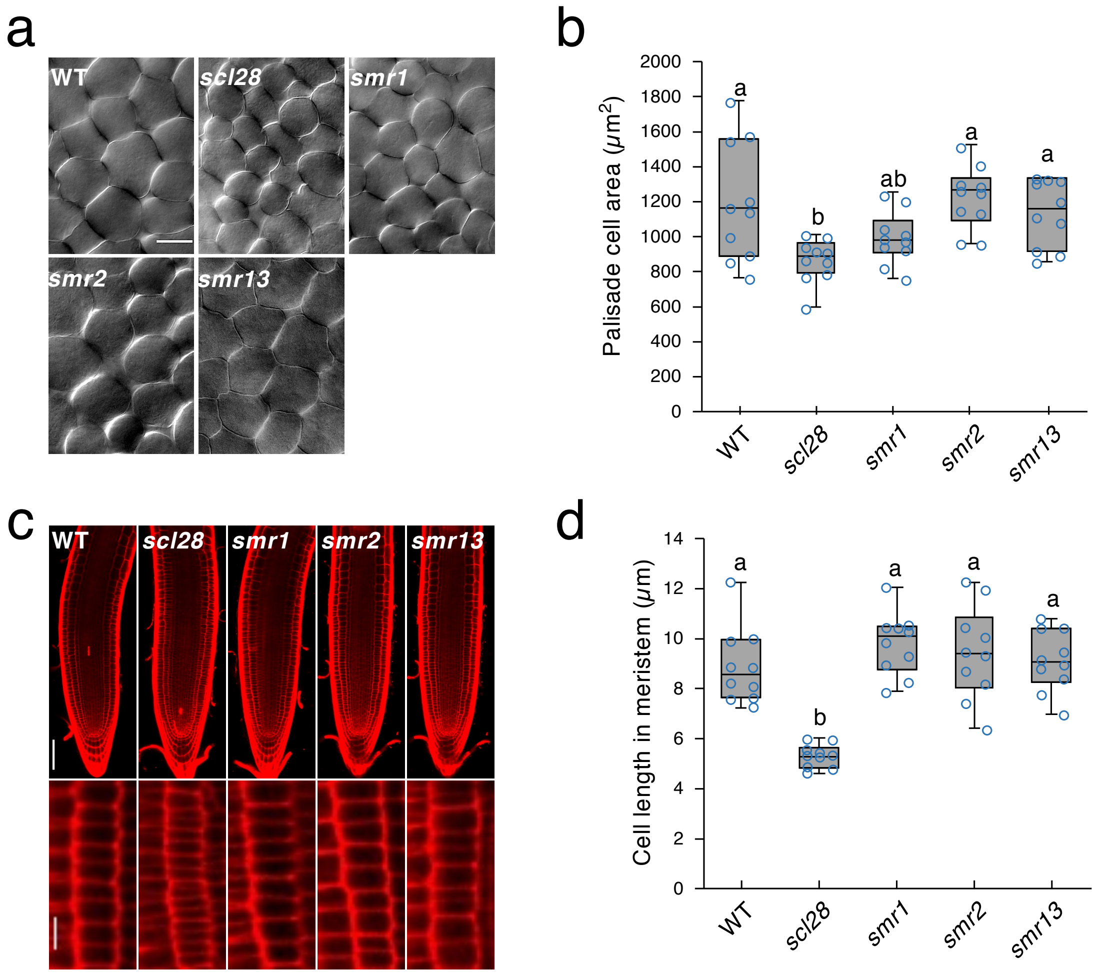

Supplementary Figure 18

Cell size phenotype of *smr1*, *smr2*, and *smr13* plants.

(a) First leaf pairs from plants at 22 DAS with indicated genotypes were cleared and observed with DIC microscopy. Scale bar indicates 30 μm.

(b) Quantification of palisade cell area in first leaf pairs from plants with indicated genotypes. Boxplot was generated using data collected from leaves of 10 different plants, in each of which more than 40 cells were analyzed (midline = median, box = IQR, whiskers = 1.5 × IQR). Different letters above boxplots indicate significant differences based on one-way ANOVA and Tukey's test, $P < 0.05$.

(c) Root meristems from plants at 7 DAS with indicated genotypes were stained by PI and observed with LSCM. Magnified views of cortical cell files are shown in lower panels. Scale bars indicate 50 μm (upper) and 15 μm (lower).

(d) Quantification of cell length in cortical cell files from plants with indicated genotypes. Boxplot was generated using data collected from roots of 10 different plants, in each of which more than 50 cells were analyzed (midline = median, box = IQR, whiskers = 1.5 × IQR). Different letters above boxplots indicate significant differences based on one-way ANOVA and Tukey's test. $P < 0.05$.

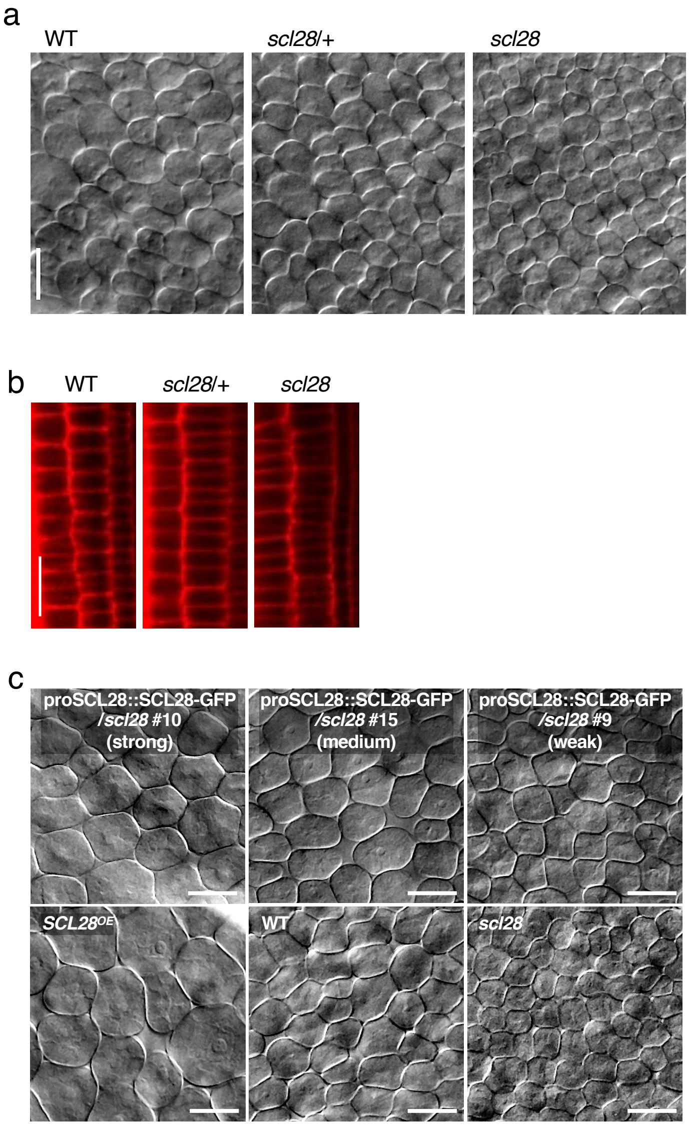

Supplementary Figure 19

Cell size phenotype of plants heterozygous for *scl28* and those with moderately increased *SCL28* expression.

(a) Images of palisade cells from WT plants and those heterozygous (*scl28*/+) or homozygous (*scl28*) for *scl28*. DIC microscopy observations were made for cleared first leaf pairs from plants at 10 DAS. Scale bar indicates 25 μm. Quantitative analysis of cell size is shown in Fig. 8b.

(b) Images of cortical cells in root meristems from WT, *scl28*/+, and *scl28* plants. Meristems of primary roots were stained by PI and observed with LSCM. Scale bar indicates 25 μm. Quantitative analysis of cell size is shown in Fig. 8c.

(c) Images of palisade cells from different transgenic lines of proSCL28::SCL28-GFP under *scl28* background with various expression levels (strong, medium, and weak). For comparison, WT, *scl28* and *SCL28*^{OE} plants were also analyzed. DIC microscopy observations were made for cleared first leaf pairs from plants at 10 DAS with indicated genotypes. Scale bar indicates 20 μm. Quantitative analysis of cell size is shown in Fig. 8d.

Supplementary Table 1

Primers used in this study

Primer name	Purpose	Sequence
AtE1M gene-5/CACC	Construction of proSCL28::GUS and proSCL28::SCL28-GFP	CACCTTTGTCACGGCTTTAAACGCTTC
AtE1M gene-3	Construction of proSCL28::SCL28-GFP	AATATAATGGGCCGACCTCATAC
AtE1Mp3-1	Construction of proSCL28::GUS	AAGTCGACAACCCAATTCAAGAGATGGTCTAC
AtE1Mpro_dMSA5	Site-directed mutagenesis of proSCL28::GUS	AAATTGGTGACCAATTGTGAATTGTCGGAGAAATATGACCAATGGGAG
AtE1Mpro_dMSA6	Site-directed mutagenesis of proSCL28::GUS	AATTGGTCACCAATTTTCTACCAATTGATTTTAAAGATCCATTGGCTC
AtSMOS1 CDS-F1/CACC	Construction of pro35S::AtSMOS1	CACCATGGCGTCGGTGTCGTCGTCGGAT
AtSMOS1 CDS-R1wStop	Construction of pro35S::AtSMOS1	TCATTTCTCTTGTGGGAGGTA
SMR2p-Sma1-F	Construction of proSMR2::LUC	CACCCCCGGGAACTCCTTCGGCATCTTTGTTT
SMR2p-BamH1-R	Construction of proSMR2::LUC	CCCGGATCCGGTCACATGGATGTGAAAGTTT
AtE1M-5	Construction of proRPS5A::SCL28-GFP	CACCATCTCTTGAATTGGGTTAGGTAG
GFPstop-R	Construction of proRPS5A::SCL28-GFP	TTACTTGTACAGCTCGTCCATGCCG
AtSMOS1 gene attB1_F	Construction of proAtSMOS1::AtSMOS1-GFP	GGGGACAAGTTTGTACAAAAAAGCAGGCTAGACTTATGCAATACTGTTGG
AtSMOS1 gene attB2_R	Construction of proAtSMOS1::AtSMOS1-GFP	GGGGACCACTTTGTACAAGAAAGCTGGGTTCTGGGTAATAGGATTCAGTT
SMOS1gene-EGFP_Cfu_F	Construction of proAtSMOS1::AtSMOS1-GFP	GACGAGCTGTACAAGTGAGCCGTTCCCTTTAGACTTTATG
SMOS1gene-EGFP_Cfu_R	Construction of proAtSMOS1::AtSMOS1-GFP	GCCCTTGCTCACCATTTTCTCTTGTGGGAGGTAGCTG
AtE1M_over insertion_qF	qRT-PCR for SCL28	GCCTTCAAACAGAGGAATCTGG
AtE1M_over insertion_qR	qRT-PCR for SCL28	TTGGTAAGCTTCATGGGAGCTC
CYCB1;2-qFW	qRT-PCR for CYCB1;2	GAATATGGTTCACTTCCTTGC
CYCB1;2-qRV	qRT-PCR for CYCB1;2	CTGCAATGTATCAGTCCAAGC
UBQ5_F	qRT-PCR for UBQ5	CTTGAAGACGGCCGTACCCTC
UBQ5_R	qRT-PCR for UBQ5	CGCTGAACCTTTCCAGATCCATCG
KNOLLE-Q4	qRT-PCR for KNOLLE	TGATGGTTGAATCGCAAGGTGAAC
KNOLLE-Q3	qRT-PCR for KNOLLE	TGCAGTCTTCAGCTCATTAGCTC
SIM_F1	qRT-PCR for SIM	TCTTCCGACCACAAGATTCC
SIM_R1	qRT-PCR for SIM	TCTTGCAAGATCTGATGCCG
SMR1_qF3	qRT-PCR for SMR1	CACCCACATCCCAAGAAC
SMR1_qR3	qRT-PCR for SMR1	GACGGAGGAGAAGAAACG
SMR2_F3	qRT-PCR for SMR2	CAAGATTGTCCAAAGATCTTCGG
SMR2_R1	qRT-PCR for SMR2	GGCACTATTACTCCTTCGTTTC
SMR3_qF	qRT-PCR for SMR3	CGATCACAAGATTCCGGAGGTG
SMR3_qR	qRT-PCR for SMR3	CGGCTCAGATCAATCGGTATGC
SMR4_qF2	qRT-PCR for SMR4	TGGTGGTGAGAAAACGAGATCC
SMR4_qR2	qRT-PCR for SMR4	AGGCTGTGCGTAGAACAAAG
SMR5_F	qRT-PCR for SMR5	CGTGATGATTGCCGGATACC
SMR5_R	qRT-PCR for SMR5	AAAATATCCCTTCTTCGGTGGTTC
SMR6_qF2	qRT-PCR for SMR6	TTTCGATTCGGGCTTCGTTG
SMR6_qR2	qRT-PCR for SMR6	TCTTCGTTTCCTTCGCTGTC
SMR7_F1	qRT-PCR for SMR7	CAGAGAATTAGACACCGATG
SMR7_R1	qRT-PCR for SMR7	CGTGGGAGTGATACAAATTC
SMR8_qF3	qRT-PCR for SMR8	AAACCGTCGTTGAAGTGCAG
SMR8_qR2	qRT-PCR for SMR8	GGGATCAGAGTCGTGAAAACAG
SMR9_qF2	qRT-PCR for SMR9	AAAAGGTGGCGCAAAACTGC
SMR9_qR2	qRT-PCR for SMR9	GTTGACCAAGTGCGAAAACG
SMR10_qF	qRT-PCR for SMR10	GCAAAGAAGGAGCAACCGTCAAG
SMR10_qR	qRT-PCR for SMR10	CGGTGGACAAATTCTTGGCATCG
SMR11_qF	qRT-PCR for SMR11	CTGCTTCGATCTCGGATTGTGTT
SMR11_qR	qRT-PCR for SMR11	GACGAAGGAGGCGGTGTTTTAC
SMR12_qF	qRT-PCR for SMR12	GGTATGTCGGAGACGAGCTTGA
SMR12_qR	qRT-PCR for SMR12	GAGTCGGTGTCTTGAACCCATCA
SMR13_qF	qRT-PCR for SMR13	GAGTCTCCTGTAAAGATCCCAG
SMR13_R2	qRT-PCR for SMR13	TAGCTTTTGGCTTTCTCGGC
SMR14_qF1	qRT-PCR for SMR14	AACCAAACCGAGCCGAAAAG
SMR14_qR1	qRT-PCR for SMR14	GTGTTGATGTTGTTGTGTTGAGG
SMR15_qF	qRT-PCR for SMR15	AATGCGTCATCACCGGAATC
SMR15_qR2	qRT-PCR for SMR15	TGGTGGCGAAAAGAACTCTC
SMR16_qF	qRT-PCR for SMR16	GCCTTCAAACAGAGGAATCTGG
SMR16_qR	qRT-PCR for SMR16	TTGGTAAGCTTCATGGGAGCTC
KRP1 F	qRT-PCR for KRP1	CGGTGATAATGGAGTTTCGTC
KRP1 R	qRT-PCR for KRP1	TCCCGCTACAACAACAATCTAA
KRP2 F	qRT-PCR for KRP2	GGTGAAACGAAGGAAGATGG
KRP2 R	qRT-PCR for KRP2	ACAACACGAAACCGACGAA
KRP3 F	qRT-PCR for KRP3	GTGAAATGGAGGAGTTCTTTGC
KRP3 R	qRT-PCR for KRP3	GCTGAGGGGGATATCATTCA
KRP4 F	qRT-PCR for KRP4	CCCTAGGAGTAATTTGGATTCG
KRP4 R	qRT-PCR for KRP4	CACTTTCAGAAACAGAATCAGACC
KRP5 F	qRT-PCR for KRP5	AAAGTGAGATTGAAGACTTCTTTGC
KRP5 R	qRT-PCR for KRP5	TTGTCCGAGACAATGTCAAAGT
KRP6 F	qRT-PCR for KRP6	GAAACCGAAACCGAAACCTC
KRP6 R	qRT-PCR for KRP6	CCCTCACTCACTGGACTCGT

Supplementary Table 1 (Continued)

Continued

Primer name	Purpose	Sequence
KRP7 F	qRT-PCR for KRP7	GCAGGCAGAGCTTGATGACT
KRP7 R	qRT-PCR for KRP7	TCATTGACGATGTCGTAGTTGTACT
ACT2 q3	qRT-PCR for ACT2	AATCCAGCACAATACCGGTTGTAC
ACT2 q5	qRT-PCR for ACT2	TCCTCTTAACCCAAAGGCCAACAG
UBQ(-253/-32)_F	ChIP-qPCR for UBQ10 promoter	AATAAACGGCGTCAAAGTGG
UBQ(-253/-32)_R	ChIP-qPCR for UBQ10 promoter	ACGAGGACGACTAGGTCACG
pSMR2_(-567)_F	ChIP-qPCR for SMR2 promoter (distal)	GCGAAGGAGCGAATAATTCC
pSMR2_(-392)_R	ChIP-qPCR for SMR2 promoter (distal)	CGGAGAAGCTTATCCAAATTAATG
pSMR2_(-177)_F	ChIP-qPCR for SMR2 promoter (proximal)	GATTAATTCATACATGTACATTTTCGC
pSMR2_(-10)_R	ChIP-qPCR for SMR2 promoter (proximal)	GATGTGAAAGTTTCGTGGGC
SMR2_F3	ChIP-qPCR for SMR2 CDS	CAAGATTGTCCAAAGATCTTCGG
SMR2_R1	ChIP-qPCR for SMR2 CDS	GGCACTATTACTCCTTCGTTTC
pSMOS1(-300)_qF	ChIP-qPCR for AtSMOS1 promoter	CAACAACATCGTGAGTGTACC
pSMOS1(-300)_qR	ChIP-qPCR for AtSMOS1 promoter	CGTGTACGTGATCTGTTTCTGG
SMOS1_cds_qF1	ChIP-qPCR for SMR2 CDS	AGCCAACAAGAACCGTCAAC
SMOS1_cds_qR1	ChIP-qPCR for SMR2 CDS	ACAAAGGACGCCAAGGTTTG

References

Dietz, K. J., Vogel, M. O. & Viehhauser, A. AP2/EREBP transcription factors are part of gene regulatory networks and integrate metabolic, hormonal and environmental signals in stress acclimation and retrograde signalling. *Protoplasma* **245**, 3-14 (2010).

Edgar, R. C. MUSCLE: multiple sequence alignment with high accuracy and high throughput. *Nucleic Acids Res*. **32**, 1792-1797 (2004).

Goodstein, D. M., Shu, S., Howson, R., Neupane, R., Hayes, R. D., Fazo, J., Mitros, T., Dirks, W., Hellsten, U., Putnam, N. & Rokhsar, D. S. Phytozome: a comparative platform for green plant genomics. *Nucleic Acids Res*. **40** (Database issue), D1178-1186 (2112).

Kumar, S., Stecher, G., Li, M., Knyaz, C. & Tamura, K. MEGA X: Molecular Evolutionary Genetics Analysis across Computing Platforms. *Mol. Biol. Evol*. **35**, 1547-1549 (2018).

Magyar, Z., Horváth, B., Khan, S., Mohammed, B., Henriques, R., De Veylder, L., Bakó, L., Scheres, B. & Bögre, L. Arabidopsis E2FA stimulates proliferation and endocycle separately through RBR-bound and RBR-free complexes. *EMBO J*. **31**: 1480–1493 (2012).

Nowack, M. K., Harashima, H., Dissmeyer, N., Zhao, X., Bouyer, D., Weimer, A. K., De Winter, F., Yang, F. & Schnittger, A. Genetic framework of cyclin-dependent kinase function in *Arabidopsis*. *Dev. Cell* **22**, 1030-1040 (2012).

Őszi, E., Papdi, C., Mohammed, B., Petkó-Szandtner, A., Leviczky, T., Molnár, E., Galvan-Ampudia, C., Khan, S., Juez, E.L., Horváth, B., Bögre, L. & Magyar, Z. E2FB Interacts with RETINOBLASTOMA RELATED and Regulates Cell Proliferation during Leaf Development. *Plant Physiol*. **182**, 518-533 (2020).

Saitou, N. & Nei, M. The neighbor-joining method: a new method for reconstructing phylogenetic trees. *Mol. Biol. Evol*. **4**, 406-25 (1987).

Sharoni, A. M., Nuruzzaman, M., Satoh, K., Shimizu, T., Kondoh, H., Sasaya, T., Choi, I. R., Omura, T. & Kikuchi, S. Gene structures, classification and expression models of the AP2/EREBP transcription factor family in rice. *Plant Cell Physiol*. **52**, 344-60 (2011).

Wang, S., Gu, Y., Zebell, S.G., Anderson, L.K., Wang, W., Mohan, R. & Dong, X. A noncanonical role for the CKI-RB-E2F cell-cycle signaling pathway in plant effector-triggered immunity. *Cell Host. Microbe* **16**, 787-794 (2014).
