# Supplementary material for: A hierarchical transcriptional network activates specific CDK inhibitors that regulate G2 to control cell size and number in Arabidopsis
Source: Nat Commun. 2022 Mar 29;13:1660. doi: 10.1038/s41467-022-29316-2 (PMC8964727; doi:10.1038/s41467-022-29316-2)
Supplement: Supplementary file 3 — Description of Additional Supplementary Files [file 41467_2022_29316_MOESM3_ESM.pdf]

Description of Additional Supplementary Files

File name: Supplementary Data 1

Description: Differentially expressed genes in *scl28*, *atsmos1* and *SCL28*^{OE} plants compared to wild type plants.

File name: Supplementary Data 2

Description: Direct target genes bound by SCL28 and AtSMOS1 as defined by ChIP-Seq analysis.
