# Supplementary material for: A hierarchical transcriptional network activates specific CDK inhibitors that regulate G2 to control cell size and number in Arabidopsis
Source: Nat Commun. 2022 Mar 29;13:1660. doi: 10.1038/s41467-022-29316-2 (PMC8964727; doi:10.1038/s41467-022-29316-2)
Supplement: Supplementary file 6 — Reporting Summary [file 41467_2022_29316_MOESM6_ESM.pdf]

nature portfolio

Corresponding author(s): Masaki Ito

Last updated by author(s): January 25, 2022

Reporting Summary

Nature Portfolio wishes to improve the reproducibility of the work that we publish. This form provides structure for consistency and transparency in reporting. For further information on Nature Portfolio policies, see our Editorial Policies and the Editorial Policy Checklist.

Statistics

For all statistical analyses, confirm that the following items are present in the figure legend, table legend, main text, or Methods section.

n/a	Confirmed	
☐	☒	The exact sample size (*n*) for each experimental group/condition, given as a discrete number and unit of measurement
☐	☒	A statement on whether measurements were taken from distinct samples or whether the same sample was measured repeatedly
☐	☒	The statistical test(s) used AND whether they are one- or two-sided<br>*Only common tests should be described solely by name; describe more complex techniques in the Methods section.*
☒	☐	A description of all covariates tested
☒	☐	A description of any assumptions or corrections, such as tests of normality and adjustment for multiple comparisons
☐	☒	A full description of the statistical parameters including central tendency (e.g. means) or other basic estimates (e.g. regression coefficient) AND variation (e.g. standard deviation) or associated estimates of uncertainty (e.g. confidence intervals)
☐	☒	For null hypothesis testing, the test statistic (e.g. *F*, *t*, *r*) with confidence intervals, effect sizes, degrees of freedom and *P* value noted<br>*Give P values as exact values whenever suitable.*
☒	☐	For Bayesian analysis, information on the choice of priors and Markov chain Monte Carlo settings
☒	☐	For hierarchical and complex designs, identification of the appropriate level for tests and full reporting of outcomes
☒	☐	Estimates of effect sizes (e.g. Cohen's *d*, Pearson's *r*), indicating how they were calculated

Our web collection on statistics for biologists contains articles on many of the points above.

Software and code

Policy information about availability of computer code

Data collection	Numerical computing and statistical analysis were conducted using Ecxel (Microsoft 365) and R (version 4.1.2)<br>qPCR was performed with StepOnePlus Real-Time PCR Systems (Applied Biosystems) and associated software.<br>Cell size and number in mesophyll and root meristem was measured and collected by Fiji (imageJ).<br>Ploidy data was collected using CyStain UV precise P kit (Sysmex) and associated software.
Data analysis	RNA-Seq data analysis was performed with Bowtie and edgeR. For microarray data analysis, we used Microarray Suite ver. 5 (Affymetrix) and GeneSpring 7.1 (Agilent Technologies). ChIP-Seq data analysis was conducted with FASTQC, Trimmomatic-0.38, Bowtie2 v2.3.5, macs2 2.2.7.1, samtools v1.9, BEDTOOLS INTERSECT, IGB, HOMER and NGSplot. Gene ontology enrichment analysis was conduced using Panther (http://pantherdb.org).

For manuscripts utilizing custom algorithms or software that are central to the research but not yet described in published literature, software must be made available to editors and reviewers. We strongly encourage code deposition in a community repository (e.g. GitHub). See the Nature Portfolio guidelines for submitting code & software for further information.

Data

Policy information about availability of data

All manuscripts must include a data availability statement. This statement should provide the following information, where applicable:

- Accession codes, unique identifiers, or web links for publicly available datasets
- A description of any restrictions on data availability
- For clinical datasets or third party data, please ensure that the statement adheres to our policy

The main data supporting the finding of this study are available within the article and its Supplementary Information files. RNA-Seq data for WT, scl28, and atsmos1

can be accessed from the DDBJ database under accession number DRA012786 [https://ddbj.nig.ac.jp/resource/sra-submission/DRA012786]. ChIP-Seq data of SCL28 and AtSMOS1 can be accessed at Gene Expression Omnibus database under accession number GSE183209 [https://www.ncbi.nlm.nih.gov/geo/query/acc.cgi?acc=GSE183209]. Arabidopsis mutants and transgenic lines, as well as plasmids generated in this study are available from the corresponding author upon reasonable request. Source data are provided with this paper.

Field-specific reporting

Please select the one below that is the best fit for your research. If you are not sure, read the appropriate sections before making your selection.

[x] Life sciences   [ ] Behavioural & social sciences   [ ] Ecological, evolutionary & environmental sciences

For a reference copy of the document with all sections, see nature.com/documents/nr-reporting-summary-flat.pdf

Life sciences study design

All studies must disclose on these points even when the disclosure is negative.

Sample size	Sample sizes were chosen based on the experimental variability and amount of samples required for statistical analysis. Sample sizes are indicated in the Figure legends and main text.
Data exclusions	No data were excluded from the study.
Replication	Numbers of replicates were started in the figure legends and Method section.
Randomization	Plant samples used in this study were genetically homogeneous and of the same age under same growth condition. When sampling, plants were randomly picked up.
Blinding	Blinding was not relevant to our study.

Reporting for specific materials, systems and methods

We require information from authors about some types of materials, experimental systems and methods used in many studies. Here, indicate whether each material, system or method listed is relevant to your study. If you are not sure if a list item applies to your research, read the appropriate section before selecting a response.

Materials & experimental systems

n/a	Involved in the study	
[ ]	[x]	Antibodies
[x]	[ ]	Eukaryotic cell lines
[x]	[ ]	Palaeontology and archaeology
[x]	[ ]	Animals and other organisms
[x]	[ ]	Human research participants
[x]	[ ]	Clinical data
[x]	[ ]	Dual use research of concern

Methods

n/a	Involved in the study	
[ ]	[x]	ChIP-seq
[x]	[ ]	Flow cytometry
[x]	[ ]	MRI-based neuroimaging

Antibodies

Antibodies used	Antibodies against GFP that is commercially available. (Abcam, ab290). For each IP, 1 µg of antibody was used.
Validation	Anti-GFP antibody (ab290) is a highly versatile antibody that gives a stronger signal than other anti-GFP antibodies available. On Western blot the antibody detects the GFP fraction from cell extracts expressing recombinant GFP fusion proteins and has also been shown to be useful on mouse sections fixed with formalin. In Immunocytochemistry, the antibody gives a very good signal on recombinant YES-GFP chimeras expressed in COS cells (McCabe et al. 1999 and figure below). It is routinely used in Immunoprecipitation (IP) and IP-Western protocols and has been used successfully in HRP Immunohistochemistry at 1:200 on whole-mount mouse embryos.

ChIP-seq

Data deposition

[x] Confirm that both raw and final processed data have been deposited in a public database such as GEO.

[x] Confirm that you have deposited or provided access to graph files (e.g. BED files) for the called peaks.

Data access links	To review GEO accession GSE183209: [https://www.ncbi.nlm.nih.gov/geo/query/acc.cgi?acc=GSE183209]

[illegible]

Data access links
May remain private before publication.

Go to https://www.ncbi.nlm.nih.gov/geo/query/acc.cgi?acc=GSE183209

Files in database submission

PROCESSED DATA FILES
ChIPseq_E1M-GFP_rep1_s3norm.bw
ChIPseq_E1M-GFP_rep2_s3norm.bw
ChIPseq_SNOS1-GFP_rep1_s3norm.bw
ChIPseq_SNOS1-GFP_rep2_s3norm.bw
ChIPseq_Input_rep1_s3norm.bw
E1M-GFP_q0.05_peaks_rep1.narrowPeak.gz
E1M-GFP_q0.05_peaks_rep2.narrowPeak.gz
SNOS1-GFP_q0.05_peaks_rep1.narrowPeak.gz
SNOS1-GFP_q0.05_peaks_rep2.narrowPeak.gz
input_q0.05_peaks.narrowPeak.gz
RAW FILES
E1M-GFP.fastq.gz
SNOS1-GFP.fastq.gz
E1M_GFP_rep2_S2_R1_001.fastq.gz
SNOS1_GFP_rep2_S1_R1_001.fastq.gz
Input.fastq.gz

Genome browser session
(e.g. UCSC)

no longer applicable

Methodology

Replicates

Two biological replicates

Sequencing depth

Illumina NextSeq 500 75bp single end
Total reads and uniquely mapped reads, respectively, are as follows: 25971914 and 4174229 for SCL28 rep1, 72832400 and 11860759 for SCL28 rep2, 22036948 and 3638925 for AtSMOS1 rep1, and 26133321 and 4405981 for AtSMOS1 rep2.

Antibodies

Anti-GFP (Abcam, ab290). For each IP, 1 µg antibody was used.

Peak calling parameters

Read mapping: Trimmed reads data were mapped using bowtie2 v 2.3.5 with the following setting "bowtie2 --very-sensitive" against the genome of TAIR10.
Filtering step: Mapped reads were filtered with samtools v.1.9 with the command "samtools view -h -b -q 30 "mapping_quality >= 30"
Duplicate filtering: duplicated reads were removed with samtools v.1.9 with the command "samtools fixmate -m and samtools markdup -r "
Peak calling: peaks of read density were called with macs2 2.2.7.1 with the command "macs2 callpeak --broad -t sample.bam -c Input.bam -g 7.8e8 -q 0.05 --extsize 150 --bw 500 -B -n --outdir "

Data quality

Adapters trimming: Sequencing reads were trimmed with trimmomatic with the following command "java -jar trimmomatic-0.38.jar SE $input $output ILLUMINACLIP:TruSeq3-SE.fa:2:30:10 LEADING:5 TRAILING:5 MINLEN:30"

Software

Trimmomatic-0.38, bowtie2 v 2.3.5 , samtools v.1.9, macs2 2.2.7.1, FASTQC, BEDTOOLS INTERSECT, Integrated Genome Browser (IGB), HOMER and NGSplot.
